# Assessing the evidence for health benefits of low-level weight loss: a systematic review

**DOI:** 10.1038/s41366-024-01664-7

**Published:** 2024-11-01

**Authors:** Disha Dhar, Jessica Packer, Semina Michalopoulou, Joana Cruz, Claire Stansfield, Russell M. Viner, Oliver T. Mytton, Simon J. Russell

**Affiliations:** 1 Great Ormond Street Institute of Child Health, University College London, London, UK; 2 Institute of Education, University College London, London, UK

**Keywords:** Weight management, Health policy, Epidemiology

## Abstract

Individuals with excess weight are at a higher risk for various physical and mental health conditions. Interventions targeting weight loss can improve health, with modest weight loss of five to ten percent of body weight often considered clinically meaningful for enhancing health outcomes. However, the benefits of achieving low-level weight loss ( < 5% body weight) are poorly understood. We aimed to systematically review relevant literature and synthesise the evidence that assessed the potential health benefits of losing less than five percent body weight. We searched seven academic databases and included studies in any language, from any country, with no time constraints. We included any intervention studies that assessed the impact of less than five percent weight loss on any measured physical or mental health markers or indices. 70 studies from 68 articles were included, with study participants ranging from 14 to 10,742. In total, 137 health markers were assessed, categorised into metabolic markers (*n* = 42), cardiovascular markers (*n* = 32), anthropometric measures (*n* = 19), quality of life indices (*n* = 10), inflammatory biomarkers (*n* = 10), renal and hepatic markers (*n* = 9), psychosocial and behavioural measures (*n* = 8), pulmonary function (*n* = 3), total mortality (*n* = 2), ovulatory function (*n* = 1), and muscle strength (*n* = 1). Overall, 60% of studies reported improvements, 37% found no change or mixed results, and 3% observed a worsening of health markers or indices. Based on the available data, 87% of participants (*n* = 15,839) in the studies reported improvements in health markers or indices as a result of low-level weight loss. Our findings suggest that low-level weight loss can lead to various health benefits and challenges the conventional threshold for effective weight loss.

**Preregistration** The review protocol was pre-registered with PROSPERO (CRD42023406342)

## Introduction

Individuals with excess weight, compared to those with a healthy weight, are at an increased risk for many diseases and chronic health conditions including cardiovascular diseases, type 2 diabetes, some types of cancer, anxiety and depression [1–6]. Such comorbidities can result in reduced mobility, chronic pain, and diminished quality of life [7–10]. Obesity is associated with psychosocial difficulties, including lower self-esteem, heightened stress levels, eating disorders, as well as increased vulnerability to mental health disorders [9, 11, 12]. People living with excess weight often face stigma and discrimination [13, 14], which can result in self-stigmatisation, isolation and self-devaluation [14, 15]. Obesity is also associated with substantial social and economic consequences [16]. In the United Kingdom (UK), it is estimated that by 2050, overweight and obesity will cost the National Health Service £10 billion per year, with wider costs to society and business projected to reach £49.9 billion per year [17].

Interventions targeting weight loss can improve health and prevent obesity-related co-morbidities [18, 19]. Weight loss among individuals with excess weight can have beneficial effects on cardiovascular disease, type 2 diabetes, sleep apnoea, chronic kidney disease, hypertension, and dyslipidaemia [3, 20–23]. Guidelines from the UK and United States of America recommend achieving modest weight loss, ranging from five to ten percent, in order to yield clinically meaningful improvements in health outcomes [19, 24, 25]. As a result, weight loss of more than five percent is often cited as a key threshold for achieving clinically significant impacts and is commonly used as a target or benchmark in weight management services [3, 26–32]. While the five percent threshold provides a practical goal for weight management interventions, many participants engaging in a 12-week lifestyle intervention will not achieve this threshold [33–35]. The implications of achieving a body weight reduction of less than five percent are poorly understood. Currently, interventions resulting in less than five percent weight loss are often deemed ineffective; however, they may still offer benefits in improving health outcomes, particularly for individuals living with obesity. Exploring the potential health impacts of less than five percent weight loss, could be useful in informing policy and practice.

We aimed to systematically review and synthesise evidence that assessed the health benefits of losing less than five percent body weight on health outcomes or indicators such as, cardio-metabolic markers, wider physical markers, and psychosocial markers from controlled trials. We further aimed to describe findings by intervention type and to stratify results by baseline BMI and level of weight loss where data allowed.

## Methods

### Protocol and registration

This systematic review was registered with PROSPERO (CRD42023406342) conducted and reported according to the Preferred Reporting Items for Systematic Reviews and Meta-Analyses (PRISMA) checklist [36].

### Eligibility criteria, information sources, and search strategy

To be eligible for inclusion, studies needed to be randomised or quasi-randomised controlled trials (RCTs) or intervention studies with pre-post measures. The included exposures were weight loss interventions with lifestyle (physical activity/diet) or pharmacological components. The participant criteria were adults (18 years or older) who lost less than five percent of their body weight following an intervention. Included outcomes were any type of health measures, including physical, mental, or behavioural. The health measures of interest were broad, and searches were structured without outcome terms to ensure all relevant outcomes were captured. Findings were required to be stratified by percentage weight loss. Studies from any country, language or published at any time were included. Studies were excluded if they were non-peer reviewed articles (dissertations, conference abstracts, grey literature), if they did not include any relevant health measures, if they only presented outcomes by overall weight change (without any stratification by percentage weight loss), or if the weight loss intervention was surgical. Surgical interventions, including bariatric surgery, were excluded due to the difference in intervention intensity and the percent weight loss typically observed (typically 20 to 30% weight loss) [37–39].

Searches of the following electronic databases were conducted in March 2023: Medline (Ovid), Embase (Ovid), PsycINFO (Ovid), CINAHL (Ebsco), Cochrane Library CENTRAL, Applied Social Sciences Index and Abstracts (ProQuest), and Web of Science—Social Science Citation Index and Emerging Sources Citation Index (see Tables S1 in supplementary file for the full search strategies). The search strategy was developed by JP with oversight and input from CS (information specialist). The searches were conducted by JP and the results were firstly imported into EndNote version 20 [40] to remove duplicates, before importing into EPPI-Reviewer Version 6 software [41] to again remove duplicates and for screening and review management. Articles were double screened on title and abstract and full text by a team of reviewers (JP, SM, JC) and discrepancies were jointly reconciled.

### Assessment of quality

The Critical Appraisal Skills Programme (CASP) checklist [42] was used to assess the bias in the included studies. Bias assessment for each article was conducted independently in duplicate by a team of reviewers (JP, SM, DD) with discrepancies jointly reconciled. Studies were categorised as having a high, moderate or low risk of bias (see Table S2 in supplementary file for further details).

### Data extraction

We extracted data for participants achieving less than five percent weight loss, which may have been the whole study population or, more often, a subset of the original study population. Reported sample sizes reflect the groups relevant to our research question, often subgroups of whole study cohorts. Data extracted included study characteristics (primary author, country, year of publication), participant characteristics where possible (sample size, stratified sample size, age, baseline BMI, comorbidities), intervention characteristics (intervention type, duration, follow up), outcome details (category, measure, key finding). Corresponding authors were contacted to request additional data, where required, for the meta-analysis. Eight studies’ corresponding authors were contacted, of whom two responded with the required data. We specifically asked for mean score change, effect size measurements (e.g., standard deviation), and stratified sample sizes.

### Data synthesis

Findings across included studies were synthesised narratively. Due to the heterogeneity and constraints on the available data, meta-analysis was not possible. The data presented several constraints, such as outcomes being stratified by different weight-loss groups and values being inconsistently reported. Few studies reported the overall baseline values, while other studies reported the values by intervention group. Follow-up outcomes were also often reported only by weight-loss groups. Additionally, there were missing sample sizes and precision estimates, which further complicated the analysis.

We tabulated study characteristics and classified health markers and indices, identified across the included studies, into broader health categorises. The health markers and indices were categorised as metabolic markers, cardiovascular markers, anthropometric markers, quality of life indices, inflammatory biomarkers, renal and hepatic markers, psychosocial and behavioural measures, pulmonary function, total mortality, ovulatory function, and muscle strength.

Additionally, we classified the findings for each study into: ‘improvements’ where all studied health measures showed improvements either statistically significant or not, ‘mixed results’ where the studied health measures either showed no significant change or a mixture of improvements and declines; and ‘worsening’ where all the studied health measures that showed either statistically significant or non-significant deterioration. Table 2 showcases the overall impact of weight loss interventions on health measures of participants that loss less than five percent body weight in each study included. We considered findings by follow-up duration to assess impacts on health measures, over time. We first compared studies with less than 6 months follow up to those with 6 months or greater, and then studies with less than 12 months follow up to those with 12 months or greater.

## Results

### Study selection

**Fig. 1:**
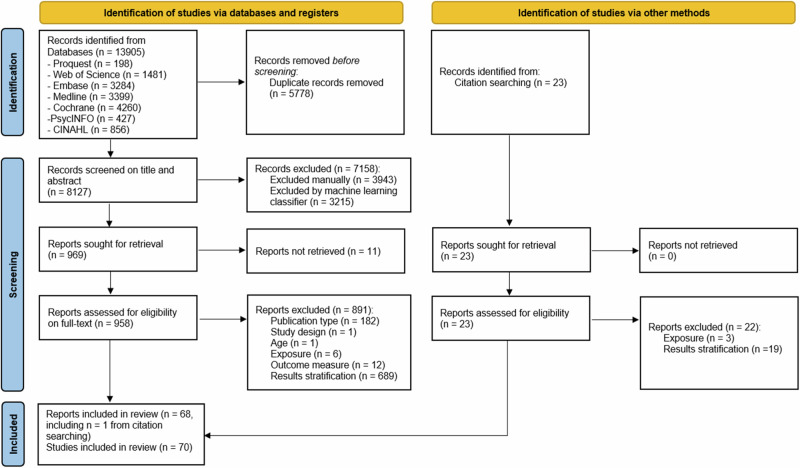
PRISMA flow chart of the review process.

Figure 1 shows the PRISMA flow chart of the search and review process. The searches resulted in 13,905 articles, of which 5778 were duplicates, leaving 8127 original articles to screen on title and abstract. After screening on title and abstract, 7158 were excluded (3943 manually and 3215 excluded by the machine learning predictive algorithm) and 969 articles were included for full-text screening, of which 11 reports were not retrieved in full-text. Application of the classifier provided articles with scores ranging from 6 to 92 which were sorted in descending order and articles with a score of 70+ were double screened; no articles were included through this process. Studies with a score between 60-69 were screened on title and abstract by one reviewer and no relevant papers were found. We excluded 891 articles that did not meet the inclusion criteria for publication type, study design, age of participants, exposure, outcome measure and stratification of results. This led to the final inclusion of 70 studies from 68 articles.

### Quality of studies

Overall, the studies were predominantly assessed as having a moderate risk of bias (*n* = 36; 53%), followed by high (*n* = 18; 26%) and low (*n* = 14; 21%). The typical issues were around randomisation methodology (see Table S2 in supplementary file for further details).

### Study description

Of the 70 included studies, the majority were randomised controlled trials (*n* = 47); the remaining study designs (*n* = 23) included before-after non-randomised intervention studies, clinical trials, cohort studies, prospective studies, and secondary analyses of trials/interventions. Most studies (*n* = 63) were conducted in high-income countries, including the USA (*n* = 34), UK (*n* = 3), Canada (*n* = 3), Japan (*n* = 3), and Australia (*n* = 2). Total study participants in the included studies ranged from 14 to 10,742. Follow-up periods ranged from six weeks to 7.4 years, with the majority of the studies (*n* = 54) having follow-up periods of 12 months or less.

Various intervention types were assessed but were predominantly lifestyle interventions (*n* = 47), with fewer studies assessing pharmacological only interventions (*n* = 2) or a combination of lifestyle and pharmacological (*n* = 21). Lifestyle interventions typically included components focussed on calorie restriction, physical activity promotion programmes, behavioural modifications, or lifestyle counselling. The most common drug utilised for pharmacological interventions were Orlistat (*n* = 6), Sibutramine (*n* = 4) and Metformin (*n* = 2).

The weight loss stratifications in included studies were most commonly less than five percent (*n* = 44), less than three percent (*n* = 7), or greater than two percent to less than five percent (*n* = 7). Studies were described based on their average baseline BMI into categories including overweight and higher (*n* = 42), obesity class-1 and higher (*n* = 9), and obesity class-2 and higher (*n* = 1), with 18 studies not reporting baseline BMI values. Study cohorts were also described based on inclusion criteria for co-morbidities, with the majority not including co-morbidities (*n* = 39), followed by metabolic syndrome (*n* = 18), diabetes (*n* = 8), and hepatic disorders (*n* = 6). Table 1 shows a descriptive summary of the included studies (see Table S5 in supplementary file for more detail).

**Table 1:** Descriptive summary of the 70 included studies.

Author	Study design	Country	Inclusion criteria	Total study participants	Intervention type	Broader health category	Overall impact on health measure/s	Overall categorisation of bias
**Abbenhardt** [43]	RCT	USA	**BMI**: ≥25 kg/m^2^	439	Lifestyle	Metabolic	Improvement	Moderate
**Ahmad** [44]	Quasi-experimental	Malaysia	**BMI**: ≥25 kg/m^2^	243	Lifestyle	Anthropometric, cardiovascular, metabolic and inflammatory biomarkers	Improvement	High
**Ahn** [45]	Open label, RCT	South Korea	**Co-morbidities:** MS and chronic kidney disease	277	Lifestyle and pharmacological	Cardiovascular, metabolic and renal/hepatic	Improvement	Moderate
**Alfaris** [90]	RCT	USA	**BMI**: ≥30 kg/m^2^ **Co-morbidities:** MS	390	Lifestyle	Quality of life	Improvement	Moderate
**Aller** [100]	RCT	Spain	**Co-morbidities:** NAFLD	36	Lifestyle and pharmacological	Renal/hepatic	Improvement	Moderate
**Annesi** [103]	Non-randomised intervention	USA	**BMI**: ≥30 kg/m^2^	128	Lifestyle	Psychosocial and behavioural	Improvement	Low
**Ashley** [81]	RCT	USA	**BMI**: ≥25 kg/m^2^	113	Lifestyle	Anthropometric, cardiovascular and metabolic	Worsening	Moderate
**Bays** - Study 1 SCALE Obesity [46]	Secondary analysis of an RCT	USA + Canada	**BMI**: ≥25 kg/m^2^	3731	Lifestyle and pharmacological	Cardiovascular	Improvement	Low
**Bays** - Study 2 SCALE Diabetes [46]	Secondary analysis of an RCT	USA + Canada	**BMI**: ≥25 kg/m^2^ **Co-morbidities:** T2D	635	Lifestyle and pharmacological	Metabolic	Improvement	Low
**Bays** - Study 3 SCALE Sleep Apnoea [46]	Secondary analysis of an RCT	USA + Canada	**BMI**: ≥25 kg/m^2^ **Co-morbidities:** Obstructive sleep apnoea	359	Lifestyle and pharmacological	Quality of life	Improvement	Low
**Campbell** [47]	RCT	USA	**BMI**: ≥25 kg/m^2^	421	Lifestyle	Metabolic	Improvement	Moderate
**Chang** [82]	Longitudinal clinical intervention	South Korea	**BMI**: ≥25 kg/m^2^ **Co-morbidities:** MS	63	Lifestyle and pharmacological	Anthropometric, cardiovascular and metabolic	Mixed results	High
**Chang** [91]	RCT	USA	**BMI**: ≥25 kg/m^2^	569	Lifestyle	Quality of life	Improvement	Moderate
**Christian** [77]	Prospective controlled trial	USA	**BMI**: ≥25 kg/m^2^ **Co-morbidities:** MS	279	Lifestyle	Anthropometric, cardiovascular, metabolic and psychosocial and behavioural	Improvement	Moderate
**D’Alonzo** [48]	RCT	USA	**BMI**: ≥25 kg/m^2^	206	Lifestyle	Metabolic	Improvement	Low
**Davidson** [49]	RCT	USA	**BMI**: ≥25 kg/m^2^ **Co-morbidities:** MS	2487	Lifestyle and pharmacological	Cardiovascular, metabolic and inflammatory biomarkers	Improvement	Low
**Del Ben** [50]	Non-randomised intervention	Italy	**Co-morbidities:** MS	172	Lifestyle	Cardiovascular and metabolic	Improvement	High
**Dittus** [64]	RCT	USA	**BMI**: ≥25 kg/m^2^	76	Lifestyle	Anthropometric and metabolic	Mixed results	High
**Dong** [65]	Prospective intervention	USA	**BMI**: ≥25 kg/m^2^	80	Lifestyle	Cardiovascular and metabolic	Mixed results	Moderate
**Duggan** [66]	RCT	USA	**BMI**: ≥25 kg/m^2^ **Co-morbidities:** Insufficient serum 25 (OH) D concentration	218	Lifestyle and pharmacological	Metabolic and inflammatory biomarkers	Mixed results	Moderate
**Duggan** [99]	RCT	USA	**BMI**: ≥25 kg/m^2^	439	Lifestyle	Inflammatory biomarkers	Mixed results	Low
**Falchi** [89]	RCT	Italy	**BMI**: ≥30 kg/m^2^	20	Lifestyle	Cardiovascular	Improvement	Moderate
**Georgoulis** [95]	RCT	Greece	**BMI**: ≥25 kg/m^2^ **Co-morbidities:** Moderate-to-severe obstructive sleep apnoea	180	Lifestyle	Quality of life	Improvement	Moderate
**Gomez-Huelgas** [67]	Open-label, non-randomised, intervention	Spain	**BMI**: ≥30 kg/m^2^ **Co-morbidities:** MS	115	Lifestyle	Anthropometric, cardiovascular, metabolic and inflammatory biomarkers	Mixed results	High
**Grandi** [78]	Non-randomised intervention	Brazil	**BMI**: ≥35 kg/m^2^ **Co-morbidities:** Asthma	51	Lifestyle	Anthropometric, metabolic, inflammatory biomarkers, quality of life, pulmonary function and muscle strength	Improvement	High
**Habermann** [79]	RCT	USA	**BMI**: ≥25 kg/m^2^	439	Lifestyle	Metabolic	No changes	Moderate
**Harrigan** [51]	RCT	USA	**BMI**: ≥25 kg/m^2^	100	Lifestyle	Metabolic and inflammatory biomarkers	Improvement	Low
**Höchsmann** [80]	Cluster RCT	USA	**BMI**: ≥30 kg/m^2^	803	Lifestyle	Cardiovascular and metabolic	Worsening	Moderate
**Imayama** [98]	RCT	USA	**BMI**: ≥25 kg/m^2^	439	Lifestyle	Inflammatory biomarkers	Improvement	Moderate
**Johnson** [83]	RCT	USA	NA	208	Lifestyle and pharmacological	Cardiovascular, metabolic and renal/hepatic	Mixed results	Moderate
**Jouneau** [107]	RCT	24 countries	**Co-morbidities:** Idiopathic pulmonary fibrosis	638	Pharmacological	Pulmonary function	Improvement	Low
**Kaholokula** [84]	RCT	USA	**BMI**: ≥25 kg/m^2^	100	Lifestyle	Cardiovascular and psychosocial and behavioural	Improvement	Moderate
**Kiddy** [68]	Non-randomised intervention	UK	**BMI**: ≥25 kg/m^2^ **Co-morbidities:** Polycystic ovary syndrome	24	Lifestyle	Metabolic and ovulatory function	Improvement	High
**Kolehmainen** [69]	RCT	Finland	**BMI**: ≥25 kg/m^2^ **Co-morbidities:** MS	46	Lifestyle	Anthropometric and metabolic	Improvement	Moderate
**Kolotkin** [96]	RCT	USA	**BMI**: ≥30 kg/m^2^ **Co-morbidities:** MS	926	Lifestyle and pharmacological	Quality of life	Mixed results	Moderate
**Konerman** [52]	Non-randomised intervention	USA	**Co-morbidities:** MS and NAFLD	403	Lifestyle	Cardiovascular, metabolic, renal/hepatic and psychosocial and behavioural	Improvement	High
**Kosiborod** [70]	Placebo-controlled trials	USA	**BMI**: ≥25 kg/m^2^ **Co-morbidities:** MS	1961	Lifestyle and pharmacological	Anthropometric, cardiovascular and metabolic	Mixed results	Low
**Lang** [53]	Non-randomised clinical trial	China	**BMI**: ≥25 kg/m^2^	14	Lifestyle	Anthropometric, cardiovascular, metabolic and inflammatory biomarkers	Improvement	High
**Magkos** [54]	RCT	USA	**BMI**: ≥25 kg/m^2^ **Co-morbidities:** T2D	434	Lifestyle and pharmacological	Metabolic	Improvement	Low
**Maruthur** [55]	RCT	USA	**Co-morbidities:** T2D	3041	Lifestyle and pharmacological	Metabolic	Improvement	Moderate
**Mason** [71]	RCT	USA	**BMI**: ≥25 kg/m^2^	439	Lifestyle	Metabolic	Mixed results	Moderate
**Messier** [97]	RCT	Canada	**BMI**: ≥25 kg/m^2^	137	Lifestyle	Psychosocial and behavioural, and quality of life	Mixed results	High
**Miazgowski** [56]	Non-randomised intervention	Poland	**Co-morbidities:** T2D or MS	111	Lifestyle	Anthropometric, cardiovascular, metabolic and renal/hepatic	Improvement	Moderate
**Muls** [87]	RCT	Belgium	**BMI**: ≥25 kg/m^2^ **Co-morbidities:** MS	294	Lifestyle and pharmacological	Cardiovascular	Mixed results	Low
**Muramoto** [57]	Controlled clinical trial	Japan	**BMI**: ≥25 kg/m^2^ **Co-morbidities:** MS	3480	Lifestyle	Anthropometric, cardiovascular, metabolic and renal/hepatic	Improvement	Moderate
**Nadinskaia** [85]	Noncomparative clinical trial	Russia, Kazakhstan, Uzbekistan	**Co-morbidities:** NAFLD	183	Lifestyle and pharmacological	Cardiovascular and renal/hepatic	Improvement	High
**Nagahara** [58]	Intervention	Japan	NA	5031	Lifestyle	Anthropometric, cardiovascular and metabolic	Improvement	High
**Patrick** – Study 2 [92]	Secondary analysis of clinical trial	USA	NA	1282	Pharmacological	Quality of life	Improvement	High
**Perreault** [72]	RCT	USA	**BMI**: ≥25 kg/m^2^	2161	Lifestyle and pharmacological	Anthropometric, cardiovascular and metabolic	Mixed results	High
**Poppitt** [88]	RCT	UK	**BMI**: ≥25 kg/m^2^	46	Lifestyle	Anthropometric and cardiovascular	Mixed results	Moderate
**Rintamaki** [59]	Cohort	Finland	**Co-morbidities:** MS, CVD or gestational diabetes	8353	Lifestyle	Cardiovascular, metabolic and total mortality	Improvement	Low
**Rock** [73]	RCT	USA	**BMI**: ≥25 kg/m^2^	258	Lifestyle	Metabolic	Mixed results	Moderate
**Rusu** [101]	RCT	Romania	**BMI**: ≥25 kg/m^2^ **Co-morbidities:** Chronic hepatitis C	120	Lifestyle	Renal/hepatic	Improvement	Moderate
**Scott** [108]	RCT	Australia	**BMI**: ≥25 kg/m^2^ **Co-morbidities:** Asthma	46	Lifestyle	Pulmonary function	Mixed results	High
**Sharma** [86]	RCT	Canada	**Co-morbidities:** CVD or T2D	10,742	Lifestyle and pharmacological	Cardiovascular	Improvement	Moderate
**Sheng** [104]	RCT	USA	**BMI**: ≥25 kg/m^2^	96	Lifestyle	Psychosocial and behavioural	Mixed results	High
**Shirai** [60]	RCT	Japan	**Co-morbidities:** T2D	240	Lifestyle	Cardiovascular and metabolic	Mixed results	Moderate
**Smith** [117]	RCT	USA and Sweden	**BMI**: ≥25 kg/m^2^	123	Lifestyle and pharmacological	Anthropometric	Mixed results	Low
**Spurny** [74]	RCT	Germany	**BMI**: ≥25 kg/m^2^	137	Lifestyle	Metabolic	Mixed results	Moderate
**St. George** [102]	RCT	Australia	**Co-morbidities:** NAFLD or chronic hepatitis C	185	Lifestyle	Renal/hepatic	Improvement	Moderate
**Strelitz** [61]	Cohort analysis -following a cluster-randomised trial	UK	**Co-morbidities:** T2D	725	Lifestyle and pharmacological	Cardiovascular, metabolic and total mortality	Improvement	Low
**Swift** [75]	RCT	USA	**BMI**: ≥25 kg/m^2^	464	Lifestyle	Anthropometric, cardiovascular and metabolic	Mixed results	Moderate
**Swift** [76]	RCT	USA	**BMI**: ≥25 kg/m^2^ **Co-morbidities:** MS	163	Lifestyle	Anthropometric, cardiovascular and metabolic	Mixed results	Moderate
**Thibault** [62]	Secondary analysis of two prospective studies	Canada	**BMI**: ≥25 kg/m^2^ **Co-morbidities:** MS	84	Lifestyle	Anthropometric and metabolic	Mixed results	High
**Tseng** [105]	Non-randomised intervention	Taiwan	NA	189	Lifestyle	Psychosocial and behavioural	Improvement	Moderate
**Vasiljevic** [93]	Non-randomised intervention	Serbia	**BMI**: ≥30 kg/m^2^	135	Lifestyle	Quality of life	Improvement	Moderate
**Vetter** [63]	RCT	USA	**BMI**: ≥30 kg/m^2^ **Co-morbidities:** MS	390	Lifestyle and pharmacological	Cardiovascular, metabolic and inflammatory biomarkers	Mixed results	Moderate
**Wing** [94]	RCT	USA	**BMI**: ≥25 kg/m^2^ **Co-morbidities:** Urinary incontinence	338	Lifestyle	Quality of life	Improvement	Low
**Wing** [26]	RCT	USA	**BMI**: ≥25 kg/m^2^	5145	Lifestyle	Cardiovascular and metabolic	Improvement	Moderate
**Wu** [106]	Non-randomised intervention	Taiwan	NA	119	Lifestyle and pharmacological	Psychosocial and behavioural	No change	High

*CVD* Cardiovascular disease, *T2D* Type 2 diabetes, *MS* Metabolic syndrome, *NAFLD* non-alcoholic fatty liver disease, *RCT* Randomised controlled trial, *USA* United States of America, *UK* United Kingdom.

While statistically significant improvements were highlighted (Table 2), non-significant improvements were classified as improvements. Low levels of weight loss, such as a 0–2% reduction in body weight, can lead to small improvements in health outcomes that may not reach statistical significance, particularly in small studies.

**Table 2:** Overall impact on health measures in included studies (*n* = 70) and participants that lost less than 5% body weight.

Overall impact on health measures	*Num. of studies (n* = *70)*	% studies	N/% of participants that lost less than 5% body weight (*n* = 18,287*)	Studies that found statistical significance (*n* = 36)
Improvements in health measures	42	60%	15,839* (86.6%)	21
Worsening in health measures	2	3%	20* (0.1%)	2
Studies that observed no-significant changes	2	3%	103 (0.6%)	–
Studies that observed mixed changes	24	34%	2325* (12.7%)	13

^*^Not all included studies provided a sample size for the <5% body weight loss category. Thus, the number may be underrepresented.

A total of 201 unique health markers and indices were reported across the 70 included studies (refer to Table S3 in the supplementary file for a detailed list). A total of 11 health categories classified health measures, including health markers and indices. These categories were reported a total of 137 times across the 70 studies (see Table 3): metabolic markers (*n* = 42), cardiovascular markers (*n* = 32), anthropometric measures (*n* = 19), quality of life indices (*n* = 10), inflammatory biomarkers (*n* = 10), renal and hepatic markers (*n* = 9), psychosocial and behavioural measures (*n* = 8), pulmonary function (*n* = 3), total mortality (*n* = 2), ovulatory function (*n* = 1), and muscle strength (*n* = 1).

**Table 3:** Impact of low-level weight loss on each health category within the 70 included studies (*n* = 137).

Health category	Improvement (%)	Worsening (%)	Mixed results (%)	No change (%)	Total health categories across included studies (*n* = 137)	Number of participants (loss < 5% body weight)
Metabolic	22 (52%)	3 (7%)	13 (31%)	4 (10%)	42	9389*
Cardiovascular	18 (56%)	1 (3%)	8 (25%)	5 (16%)	32	13,139*
Anthropometric	17 (89%)	–	–	2 (11%)	19	5004
Quality of life	6 (60%)	2 (20%)	2 (20%)	–	10	1222
Inflammatory biomarkers	6 (60%)	–	3 (30%)	1 (10%)	10	795*
Renal and hepatic	9 (100%)	–	–	–	9	1783*
Psychosocial and behavioural	4 (50%)	–	1 (12%)	3 (38%)	8	702
Pulmonary function	1 (33%)	1 (33%)	–	1 (33%)	3	774
Total mortality	–	1 (50%)	–	1 (50%)	2	642
Ovulatory function	1 (100%)	–	–	–	1	11
Muscle strength	–	–	–	1 (100%)	1	23

^*^Not all included studies provided a sample size for the <5% body weight loss category. Thus, the number may be underrepresented.

### Metabolic markers

Of the 70 studies included, metabolic markers were reported in 42 studies. Sub-sample sizes were reported for 37 of the 42 studies, with a total of 9389 participants. The most frequently reported metabolic markers were fasting plasma glucose (FPG), fasting insulin, Homoeostatic Model Assessment of Insulin Resistance (HOMA-IR), haemoglobin A1c (HbA1c), and adiponectin (APN). A total of 52 unique metabolic outcomes and indices were identified across all studies (see Table S3 in the supplementary file for detailed list of metabolic makers and indices). 22 studies (52%; *n* = 7980*) demonstrated improvements [26, 43–63], 13 studies (31%; *n* = 1006 participants*) showed mixed results [64–76], while four studies (10%; *n* = 334*) indicated no change [77–80] and three studies (7%; *n* = 69) indicated a worsening of outcomes or indices [81–83]. A robust example is a 12-month RCT, with a sample size of 2161, that assessed the impact of weight loss on various metabolic markers and found mixed results [72]. Within the <3% weight loss group there was a non-significant decrease in FPG, 2-h glucose, insulin, and HOMA-IR for both men and women; and non-significant decrease in A1C among men only. However, there was a non-significant increase in carbohydrate‐to‐insulin ratio among women and a non-significant decrease among men [72].

### Cardiovascular markers

Cardiovascular markers were reported in 32 studies (*n* = 13,139). Cardiovascular markers included total cholesterol (TC), triglycerides (TG), high-density lipoprotein cholesterol (HDL-C), low-density lipoprotein cholesterol (LDL-C), as well as systolic and diastolic blood pressure (BP). A total of 18 unique cardiovascular outcomes and indices were identified across all studies (see Table S3 in the supplementary file for detailed list of cardiovascular makers and indices). Among these studies, 18 (56%; *n* = 11,233*) found overall improvements [26, 44–46, 49, 52, 53, 56–58, 60, 61, 63, 65, 77, 84–86], eight (25%; *n* = 1362*) presented mixed results [67, 70, 72, 75, 76, 83, 87, 88], while five (16%; *n* = 519*) indicated no change [50, 59, 80, 81, 89], and one (3%; *n* = 25) showed a worsening of health markers and indices [82]. Some studies disaggregated results further, a robust and high-quality 6-week RCT investigating (*n* = 4198) the impact of weight loss on blood pressure gave mixed results for those losing between 0% and 2.5% and those losing 2.5% and 5% body weight [86]. In the 2.5–5% weight loss group there was a significant decrease in both systolic BP and diastolic BP for all patients. While, for the 0–2.5% weight loss group there was a significant decrease in systolic BP and diastolic BP for all participants after removing those classified as having high-normal BP at baseline and taking one anti-hypertensive medication [86].

### Anthropometric markers

Anthropometric markers were assessed in 19 studies, with a total of 5004 participants. Waist circumference (*n* = 15) was the most frequently reported marker, and included markers such as body fat percentage, hip circumference, and mass (kg). A total of 15 unique anthropometric outcomes and indices were identified across all studies (see Table S3 in the supplementary file for detailed list of anthropometric makers and indices). Among these studies, 17 (89%; *n* = 4767) found overall improvements in anthropometric outcomes [44, 53, 56–58, 62, 64, 67, 69, 70, 72, 75, 76, 78, 82, 88], while two studies (11%; *n* = 237) found no change [77, 81].

### Quality of life indices

Quality of life indices were assessed in 10 studies, with a total of 1222 participants. The most common indices were standardised measures of sleep duration and quality (Pittsburgh Sleep Quality Index), mood (Patient Health Questionnaire-8), asthma-related quality of life (Asthma Quality of Life Questionnaire), impact of weight on quality of life (IWQOL-Lite) and EQ-5D scores (EuroQol-5 Dimension scores). A total of 27 unique quality of life indices were identified across all studies (see Table S3 in the supplementary file for detailed list of quality of life indices). Six studies (60%; *n* = 825) found improvements in indices [46, 90–94], two studies (20%; *n* = 361) presented mixed results [95, 96], and two (20%; *n* = 36) studies indicated a worsening of indices [78, 97].

### Inflammatory biomarkers

Inflammatory biomarkers were assessed in 10 studies. Sub-sample sizes were reported in eight of the ten studies, with a total of 795 participants. The most common inflammatory biomarkers assessed were C-reactive protein (CRP), tumour necrosis factor alpha (TNF-α) and various interleukins. A total of 18 unique inflammatory outcome and indices were identified across all studies (see Table S3 in the supplementary file for detailed list of inflammatory makers and indices). Among these, six studies (60%; *n* = 670 participants; one study did not report sub-sample size) demonstrated improvements [44, 49, 51, 53, 63, 98], three studies (30%; *n* = 102 participants; one study did not report sub-sample size) presented mixed results [66, 67, 99], and one (10%; *n* = 23 participants) study indicated no change in the outcomes or indices [78].

### Renal and hepatic markers

A total of nine studies assessed renal and hepatic markers, sub-sample sizes were reported for eight of the studies, with a total of 1783 participants. Alanine transaminase (ALT), aspartate transaminase (AST), gamma-glutamyl transferase (GGT) and uric acid were the most common markers assessed in the renal and hepatic category. A total of 20 unique renal and hepatic outcomes were identified across all studies (see Table S3 in the supplementary file for detailed list of renal and hepatic makers and indices). All studies reported improvements [45, 52, 56, 57, 83, 85, 100–102]. A high quality 12-month controlled clinical trial (overall *n* = 3480), assessed the impact of weight loss on various renal and hepatic markers, including ALT, AST, GGT and uric acid and within the <3% weight reduction group found all markers improved [57].

### Psychosocial and behavioural markers

Eight studies assessed psychosocial and behavioural markers, comprising a total of 702 participants. Within the psychosocial and behavioural category, the most frequently reported markers included, physical activity-related self-regulation and self-efficacy, eating behaviours, and coping mechanisms. A total of 38 unique psychosocial and behavioural indices were identified across all studies (see Table S3 in the supplementary file for detailed list of psychosocial and behavioural makers and indices). Among these, four studies (50%; *n* = 181 participants) found improvements in markers [84, 103–105], three studies (38%; *n* = 508) reported no change [52, 77, 106] and one study (12%; *n* = 13 participants) reported mixed results [97].

### Other markers

Other health categories included pulmonary function (*n* = 3), total mortality (*n* = 2), ovulatory function (*n* = 1) and muscle strength (*n* = 1). For studies assessing pulmonary function, with a total of 774 participants, one study found improvements [107], one indicated mixed results [108], and one reported no change [78]. A total of 11 unique pulmonary function indices were identified across all studies (see Table S3 in the supplementary file for detailed list of pulmonary function makers and indices). For the studies assessing total mortality, with a total of 642 participants, neither study reported improvements, one indicated worsening [61] while the other reported no change [59]. The study assessing ovulatory function reported that one of the eight women with menstrual disturbances who lost less than five percent of their body weight noted an improvement in reproductive function [68]. The study assessing muscle strength reported no change [78].

### Further analysis

Impact on health measures did not vary by follow up duration, overall or by health category (see Table S4 in the supplementary file for further details). While the majority of health measures showed improvements across studies employing lifestyle, pharmacological, or combined interventions, there were exceptions see Table 4. There were 47 studies using only lifestyle interventions, of which 3 studies reported no change or worsening of health measures and 15 studies reported mixed results. The use of only lifestyle interventions reported no improvements in total mortality, and muscle strength. Similarly, 21 studies reporting interventions comprising both lifestyle and pharmacological interventions reported no significant changes in psychosocial and behavioural measures.

**Table 4:** The proportion of studies and intervention types that led to a change in health outcomes.

Studies by intervention type	Improvement (%)	Worsening (%)	Mixed results (%)	No change (%)
Lifestyle (*n* = 47)	29 (62%)	2 (4%)	15 (32%)	1 (2%)
Pharmacological (*n* = 2)	2 (100%)	–	–	–
Both lifestyle and pharmacological* (*n* = 21)	11 (52%)	–	9 (43%)	1 (5%)
All studies / intervention types (*n* = 70)	42 (60%)	2 (3%)	24 (34%)	2 (3%)

## Discussion

We found that weight loss of less than five percent body weight was beneficial for a range of health markers and indices. Overall, 60% of studies (comprising 87% of total participants, where reported) reported improvements in health measures, while 37% of studies (comprising 13% of participants, where reported) reported no change or mixed results, and 3% reported worsening of health measures. Low-level weight loss resulted in improvements in cardiovascular, metabolic, anthropometric, quality of life, inflammatory biomarker, renal and hepatic marker outcomes, as well as pulmonary, ovulatory function and some psychosocial, behavioural outcomes. We found no evidence that low-level weight loss improved total mortality or muscle strength. Despite low-level weight loss not being generally considered to be clinically meaningful, interventions achieving low-level weight loss could have meaningful impacts across a range of health measurements. These secondary health benefits of weight loss have implications for cost effectiveness of weight management interventions if their benefits have been under-valued.

The outcomes included in this review were varied; some were direct measures of health (e.g. mortality), some were strong predictors of future health (e.g., quality of life, BMI and muscle strength), while others had less clear prognostic value (e.g., hepatic markers, pulmonary function). Other outcomes could be considered less direct but important in their own right, such as quality of life and psychosocial measures. Of the health categories reported, cardiovascular and metabolic most frequently showed mixed results in response to low-level weight loss. In one study [88], individuals who lost <3% of their body weight showed improvements in cholesterol levels and systolic BP, while triglyceride levels and diastolic BP worsened. Conversely, when outcomes were assessed based on achieving ≥3% weight loss in the same study, nearly all parameters showed improvement, except for diastolic BP. This is supported by results from the Look AHEAD study [26], which found that weight loss of ≥2 to <5% improved some risk factors, while ≥5% to <10% led to improvements in all risk factors, and the magnitude increased with increased degree of weight loss (e.g., ≥10 to <15%, and ≥15%). Improvements were more consistently reported in anthropometric markers, renal and hepatic markers, and inflammatory biomarkers, even at low-level weight loss. This variability in cardiometabolic markers could be attributed to multiple influencing factors beyond weight loss, such as genetic predispositions, social stressors, and environmental factors [109]. Social stressors are strongly associated with cardiometabolic risk factors, thus creating a complex web of influences on cardiometabolic outcomes in weight loss interventions, making it harder to detect statistical differences at small levels of weight loss given the sample size [109].

Our findings extend previous research, which has primarily focused on cardiovascular and metabolic improvements in low-level weight loss, as well as on individuals with pre-existing cardiovascular risk factors [110, 111]. We broadened the scope to assess the impact of low-level weight loss on individuals with a variety of comorbidities, including metabolic syndrome, hepatic disorders, asthma, and obstructive sleep apnoea. This allowed us to consider quality of life indices and other health measures such as muscle strength, inflammatory biomarkers, and ovulatory function. Our findings, for example, revealed improvements in ovulatory function even with low-level weight loss, a result that aligns with existing literature showing an association between BMI with polycystic ovarian syndrome (PCOS) and infertility; weight loss within the range of 2–5% can lead to improvements in menstrual irregularities and fertility in women with PCOS [31, 32, 112]. This highlights a potentially benefit on ovulatory function following low-level weight loss.

### Implications for policy and practice

This systematic review challenges the conventional threshold for effective weight loss [31, 32, 112]. Given that lifestyle interventions for many people tend to result in low-level weight loss, our results are particularly encouraging [33–35, 113], and highlight that weight loss, which is considered not clinically meaningful, can yield meaningful health improvements. This is important message for people with lived experience of excess weight and are considering or have been referred to weight management interventions. Our findings also show the importance of considering a wide range of health measures when evaluating the efficacy of weight loss interventions, potentially reshaping how we perceive of weight management in both clinical practice and public health policy. These findings could be valuable for informing policymakers in the development of policy objectives relating to healthy weight and the evaluation of weight management services efficacy.

Our review demonstrates that low-level weight loss can positively impact not only physical health markers but also quality of life indices. Improvements in quality-of-life indices suggest that low-level weight loss may contribute to reducing broader societal costs by enhancing productivity and quality-adjusted life years. For this reason, it is important that cost-benefit and cost-effectiveness analyses of lifestyle interventions incorporate measures of well-being and quality of life, even when weight loss is minimal.

### Strengths and limitations

Strengths of our systematic review include being the first study of its kind, considering outcomes beyond cardiovascular and metabolic markers and beyond individuals with related co-morbidities. We conducted comprehensive searches across seven databases, including citation searching, with a robust assessment of study quality. We considered both statistically significant and non-significant improvements in health measures, which limits the strength of some of the findings but was important in highlighting health benefits that occurred at low levels of weight loss and in smaller studies. Small improvements in health outcomes may still be meaningful at a population level if weight loss interventions are delivered at scale. Additionally, we utilised software and machine learning for a rapid, extensive review through active learning. However, the machine learning approach does have some limitations as we excluded several studies without screening. It is possible that relevant studies were missed but following an established methodology [114] means this is highly unlikely.

We have presented the findings of this study comprehensively, systematically and in detail; however, the limited use of statistical methods to synthesise the findings is a weakness. This reflects the high heterogeneity among included studies in reported information and interventions, including variations in the stratification of results based on weight lost, participant demographics, study designs, outcome measures, and follow-up periods. These differences prevented statistical analyses, and standard comparisons via meta-analysis, limiting the generalisability of our findings. The majority of studies (77%) had follow-up periods of 12 months or less, some studies featured substantially longer follow-up periods. This necessitates further caution when extrapolating findings to longer time periods.

The data derived from subgroup analyses and stratifications were not consistent across studies. Despite this, we examined the results while considering the sample sizes of the studies to weight the findings in our synthesis. This limitation highlights the need for uniform definitions and measurements of weight loss and health outcomes. In this review, we did not attempt to differentiate outcomes based on how directly they influence health. However, we note that some outcomes (e.g. mortality) are direct health outcomes, others are strong predictors of future health (e.g. quality of life), some are risk factors for disease (e.g. blood pressure, BMI), some are measures of disease severity/process (e.g. liver function tests), while others were related to patient experience or well-being (e.g. quality of life). For some indicators, in the absence of established disease, the prognostic and patient value (e.g. liver function tests) is unclear.

### Research implications

To enable meta-analyses, future RCTs should prioritise standardised methodologies. This includes uniform definitions and measurements of weight loss and health outcomes, as well as consistent reporting of baseline characteristics and follow-up data across studies. Additionally, gathering data on the long-term impacts of low-weight loss could improve our understanding of whether these health effects sustain, even in the event of weight regain. Our findings support previous work that shows an incremental relationship between BMI and health [115], while this review suggest low-level weight loss leads to health improvements, the data shows variability, with some effects observed in some individuals and minimal or no changes in others. Identifying factors contributing to this variability, such as genetic predispositions, lifestyle factors, and metabolic profiles, would help tailor weight loss strategies. Our findings also suggest that cost-effectiveness of weight management interventions, particularly lower tier lifestyle interventions should account for the secondary health benefits of low-level weight loss.

Exploring the dose-response relationship between the extent of weight loss and health outcomes is another important area for future research. Determining how different degrees of weight loss affect various health indicators and identifying the likely graduated health benefits of increasing weight loss can inform weight management strategies and interventions. Investigating potential variations in these likely benefits by racial and ethnic groups, as well as demographic factors, could provide valuable insights. A notable gap in the current literature is the scarcity of studies that consider well-being as a secondary outcome in weight management interventions. Patient experience and well-being are increasingly being recognised as important success markers of weight management interventions [116]. Evaluations should consider patient experience, non-stigmatising approaches, and the extent to which individuals feel heard and supported throughout their weight management process as key outcomes. This could strengthen and inform the development of patient-centred approaches in weight management.

## Conclusion

The findings from this systematic review demonstrates that low-level weight loss can lead to improvements in cardiovascular, metabolic, renal and hepatic, inflammatory, ovulatory, and psychosocial measures that are likely to result in health improvements. This challenges the conventional view that weight loss above 5% body weight is necessary to be clinically meaningful. Whilst benefits may be greater at higher levels of weight loss, the findings suggest weight management services should not be overly fixated on achieving a minimum threshold of 5%. It also shows the scope for small levels of weight loss to impact on a broad range of factors, including quality of life, which will be important to communicate to people trying to lose weight and should be considered when evaluating weight management services.

To integrate these findings into clinical practice, clinicians and academics should consider a more holistic assessment of weight loss outcomes, including emphasis on quality of life, mental well-being, and psychosocial and behavioural markers. Integrating patient-centred approaches in weight management programmes is crucial, in order to improve support, and acknowledge and address the stigma of living with an unhealthy weight. Future research should prioritise standardising outcome measures and definitions to facilitate long-term tracking of health impacts and allow meta-analyses when synthesising available evidence. Implementing these changes in clinical practice and public health policy will be important in moving towards a comprehensive and effective approach to weight management.

## Supplementary information

Tables S1, Table S2, Table S3, Table S4, and Table S5
