# Supplementary material for: Assessing the evidence for health benefits of low-level weight loss: a systematic review
Source: Int J Obes (Lond). 2024 Nov 1;49(2):254–68. doi: 10.1038/s41366-024-01664-7 (PMC11805710; doi:10.1038/s41366-024-01664-7)
Supplement: Supplementary file 1 — Tables S1, Table S2, Table S3, Table S4, and Table S5 [file 41366_2024_1664_MOESM1_ESM.docx]

**Supplementary file**

**Tables S1** – Search strategies for academic databases.

Databases searched:

- OVID Medline
- OVID Embase
- OVID PsycINFO
- Cochrane Library CENTRAL
- CINAHL (Ebsco)
- Applied Social Sciences Index and Abstracts (ProQuest)
- Web of Science- social science and emerging sources

OVID Medline

| 1 | exp animals/ not humans.sh. | 5100299 |
| --- | --- | --- |
| 2 | (exp infant/ or exp child/ or exp adolescent/) not exp adult/ | 2114442 |
| 3 | (randomized controlled trial or controlled clinical trial or pragmatic clinical trial or multicenter study).pt. | 912872 |
| 4 | non-randomized controlled trials as topic/ or controlled before-after studies/ | 1766 |
| 5 | (randomis* or randomiz* or randomly or trial or multicenter or "multi center" or multicentre or "multi centre" or intervention? or controlled or (control adj group?) or (before adj5 after) or (pre adj5 post) or ((pretest or "pre test") and (posttest or "post test")) or quasiexperiment* or quasi experiment* or evaluat* or "repeated measur*").ti,ab. | 7030758 |
| 6 | ("review" or "systematic review" or comment or editorial or meta analysis or news).pt. or (systematic review or literature review).ti. | 4927222 |
| 7 | 3 or 4 or 5 | 7222410 |
| 8 | 7 not 6 | 6398829 |
| 9 | (("5" or five) adj3 ("weight loss" or "weight reduc*" or "weight decreas*" or "weight chang*")).ti,ab. | 3344 |
| 10 | (("weight loss*" adj8 stratif*) or ("weight reduc*" adj8 stratif*) or ("weight decreas*" adj8 stratif*) or ("weight chang*" adj8 stratif*)).ti,ab. | 182 |
| 11 | (("5" or five) adj3 (decrease or reduc* or loss) adj3 weight).ti,ab. | 3158 |
| 12 | (("5" or five or four or three or two or one or "4" or "3" or "2" or "1") adj2 (decrease or reduc* or loss) adj3 weight).ti,ab. | 6718 |
| 13 | ((four or three or two or one or "4" or "3" or "2" or "1") adj2 ("weight loss" or "weight reduc*" or "weight decreas*" or "weight chang*")).ti,ab. | 8032 |
| 14 | ((satisfactory or moderate or modest or low or less than) adj2 ("weight loss" or "weight reduc*" or "weight decreas*")).ti,ab. | 2491 |
| 15 | 9 or 10 or 11 or 12 or 13 or 14 | 11685 |
| 16 | (effect? Or impact? or benefit? or improvement? or improve? or relations* or associat* or risk or risks).ti. | 4445418 |
| 17 | treatment outcome/ or biomarkers/ | 1456941 |
| 18 | ("risk factor*" or (risk adj predict*) or correlation* or biomarker* or causality or associate* or "predisposing factors" or "enabling factors" or "precipitating factors" or "reinforcing factors" or "patient reported outcome*" or prognosis* or prognostic* or mortality).ti,ab. | 7049867 |
| 19 | 16 or 17 or 18 | 10442445 |
| 20 | 8 and 15 and 19 | 4071 |
| 21 | 20 not (1 or 2) | 3699 |
| 22 | 21 not ("bariatric surgery" or "gastric sleeve" or "gastric bypass").ti. | 3399 |

OVID EMBASE

| 1 | not ((exp animal/ or animal experiment/ or nonhuman/) not (exp human/ or human experiment/)) | 6757185 |
| --- | --- | --- |
| 2 | not (exp juvenile/ not exp adult/) | 2395499 |
| 3 | non-randomized controlled trials as topic/ or controlled before-after studies/ | 211779 |
| 4 | (randomis* or randomiz* or randomly or trial or multicenter or "multi center" or multicentre or "multi centre" or intervention? or controlled or (control adj group?) or (before adj5 after) or (pre adj5 post) or ((pretest or "pre test") and (posttest or "post test")) or quasiexperiment* or quasi experiment* or evaluat* or "repeated measur*").ti,ab. | 9708462 |
| 5 | (systematic review or literature review).ti. | 319471 |
| 6 | 3 or 4 | 9861155 |
| 7 | 6 not 5 | 9680713 |
| 8 | (("5" or five) adj3 ("weight loss" or "weight reduc*" or "weight decreas*" or "weight chang*")).ti,ab. | 6102 |
| 9 | (("weight loss*" adj8 stratif*) or ("weight reduc*" adj8 stratif*) or ("weight decreas*" adj8 stratif*) or ("weight chang*" adj8 stratif*)).ti,ab. | 332 |
| 10 | (("5" or five) adj3 (decrease or reduc* or loss) adj3 weight).ti,ab. | 5810 |
| 11 | (("5" or five or four or three or two or one or "4" or "3" or "2" or "1") adj2 (decrease or reduc* or loss) adj3 weight).ti,ab. | 12888 |
| 12 | ((four or three or two or one or "4" or "3" or "2" or "1") adj2 ("weight loss" or "weight reduc*" or "weight decreas*" or "weight chang*")).ti,ab. | 11134 |
| 13 | ((satisfactory or moderate or modest or low or less than) adj2 ("weight loss" or "weight reduc*" or "weight decreas*")).ti,ab. | 3603 |
| 14 | 8 or 9 or 10 or 11 or 12 or 13 | 20631 |
| 15 | (effect? Or impact? or benefit? or improvement? or improve? or relations* or associat* or risk or risks).ti. | 5227061 |
| 16 | treatment outcome/ or biomarkers/ | 1309467 |
| 17 | ("risk factor*" or (risk adj predict*) or correlation* or biomarker* or causality or associate* or "predisposing factors" or "enabling factors" or "precipitating factors" or "reinforcing factors" or "patient reported outcome*" or prognosis* or prognostic* or mortality).ti,ab. | 9531650 |
| 18 | 15 or 16 or 17 | 12964145 |
| 19 | 7 and 14 and 18 | 7508 |
| 20 | 19 not (1 or 2) | 6866 |
| 21 | 20 not ("bariatric surgery" or "gastric sleeve" or "gastric bypass").ti. | 6352 |
| 22 | Limit 21 to (article or article in press) | 3284 |

OVID PsycINFO

| 1 | (exp Animals/ or exp Animal Research/) not (exp Human Females/ or exp Human Males/) | 372643 |
| --- | --- | --- |
| 2 | ("100" or "120" or "140" or "160" or "180" or "200").ag. | 870633 |
| 3 | ("300" or "320" or "340" or "360" or "380" or "390").ag. | 2184092 |
| 4 | 2 not 3 | 548788 |
| 5 | (randomis* or randomiz* or randomly or trial or multicenter or "multi center" or multicentre or "multi centre" or intervention? or controlled or (control adj group?) or (before adj5 after) or (pre adj5 post) or ((pretest or "pre test") and (posttest or "post test")) or quasiexperiment* or quasi experiment* or evaluat* or "repeated measur*").ti,ab. | 1264572 |
| 6 | (systematic review or literature review).ti. | 37097 |
| 7 | 5 not 6 | 1243263 |
| 8 | (("5" or five) adj3 ("weight loss" or "weight reduc*" or "weight decreas*" or "weight chang*")).ti,ab. | 412 |
| 9 | (("weight loss*" adj8 stratif*) or ("weight reduc*" adj8 stratif*) or ("weight decreas*" adj8 stratif*) or ("weight chang*" adj8 stratif*)).ti,ab. | 23 |
| 10 | (("5" or five) adj3 (decrease or reduc* or loss) adj3 weight).ti,ab. | 377 |
| 11 | (("5" or five or four or three or two or one or "4" or "3" or "2" or "1") adj2 (decrease or reduc* or loss) adj3 weight).ti,ab. | 791 |
| 12 | ((four or three or two or one or "4" or "3" or "2" or "1") adj2 ("weight loss" or "weight reduc*" or "weight decreas*" or "weight chang*")).ti,ab. | 771 |
| 13 | ((satisfactory or moderate or modest or low or less than) adj2 ("weight loss" or "weight reduc*" or "weight decreas*")).ti,ab. | 246 |
| 14 | 8 or 9 or 10 or 11 or 12 or 13 | 1403 |
| 15 | (effect? or impact? or benefit? or improvement? or improve? or relations* or associat* or risk or risks).ti. | 837889 |
| 16 | treatment outcome/ or biomarkers/ | 56488 |
| 17 | ("risk factor*" or (risk adj predict*) or correlation* or biomarker* or causality or associate* or "predisposing factors" or "enabling factors" or "precipitating factors" or "reinforcing factors" or "patient reported outcome*" or prognosis* or prognostic* or mortality).ti,ab. | 1037484 |
| 18 | 15 or 16 or 17 | 1625547 |
| 19 | 7 and 14 and 18 | 483 |
| 20 | 19 not (1 or 4) | 445 |
| 21 | 20 not ("bariatric surgery" or "gastric sleeve" or "gastric bypass").ti. | 427 |

Cochrane Library CENTRAL

| 1 | MeSH descriptor: [Animals] explode all trees | 761337 |
| --- | --- | --- |
| 2 | MeSH descriptor: [Humans] explode all trees | 758651 |
| 3 | #1 NOT #2 | 2686 |
| 4 | MeSH descriptor: [Child] explode all trees | 70846 |
| 5 | MeSH descriptor: [Adult] explode all trees | 541581 |
| 6 | #4 NOT #5 | 51199 |
| 7 | MeSH descriptor: [Controlled Clinical Trials as Topic] explode all trees | 45669 |
| 8 | MeSH descriptor: [Randomized Controlled Trial] explode all trees | 113 |
| 9 | MeSH descriptor: [Randomized Controlled Trials as Topic] explode all trees | 45263 |
| 10 | MeSH descriptor: [Controlled Clinical Trial] explode all trees | 122 |
| 11 | MeSH descriptor: [Controlled Before-After Studies] explode all trees | 112 |
| 12 | MeSH descriptor: [Multicenter Study] explode all trees | 10 |
| 13 | MeSH descriptor: [Pragmatic Clinical Trials as Topic] explode all trees | 322 |
| 14 | (randomis* or randomiz* or randomly or trial or multicenter or "multi center" or multicentre or "multi centre" or intervention? or controlled or (control adj group?) or (before NEXT after) or (pre NEXT post) or ((pretest or "pre test") and (posttest or "post test")) or quasiexperiment* or quasi experiment* or evaluat* or "repeated measur*"):ti,ab | 1554329 |
| 15 | #7 OR #8 OR #9 OR #10 OR #11 OR #12 OR #13 OR #14 | 1563628 |
| 16 | ((5 or five) NEXT (weight loss or weight reduc* or weight decreas* or weight chang*)):ti,ab | 3271 |
| 17 | (("weight loss*" NEAR/3 stratif*) or ("weight reduc*" NEAR/3 stratif*) or ("weight decreas*" NEAR/3 stratif*) or ("weight chang*" NEAR/3 stratif*)):ti,ab | 38 |
| 18 | (("5" or five) NEAR (decrease or reduc* or loss) NEAR/2 weight):ti,ab | 2899 |
| 19 | ((("5" or five or four or three or two or one or "4" or "3" or "2" or "1") NEXT (decrease or reduc* or loss)) NEAR weight):ti,ab | 357 |
| 20 | ((four or three or two or one or "4" or "3" or "2" or "1") NEXT ("weight loss" or "weight reduc*" or "weight decreas*" or "weight chang*")):ti,ab | 2328 |
| 21 | ((satisfactory or moderate or modest or low or less than) NEAR ("weight loss" or "weight reduc*" or "weight decreas*")):ti,ab | 3200 |
| 22 | #16 OR #17 OR #18 OR #19 OR #20 OR #21 | 9899 |
| 23 | MeSH descriptor: [Treatment Outcome] explode all trees | 174018 |
| 24 | MeSH descriptor: [Biomarkers] explode all trees | 26628 |
| 25 | (impact? or benefit? or improvement? or improve? or relations* or associat* or risk or risks):ti | 196204 |
| 26 | ("risk factor*" or (risk NEXT predict*) or correlation* or biomarker* or causality or associate* or "predisposing factors" or "enabling factors" or "precipitating factors" or "reinforcing factors" or "patient reported outcome*" or prognosis* or prognostic* or mortality):ti,ab | 426466 |
| 27 | #23 OR #24 OR #25 OR #26 | 658936 |
| 28 | #15 AND #22 AND #27 | 4594 |
| 29 | #28 NOT (#3 OR #6) | 4538 |
| 30 | ("bariatric surgery" or "gastric sleeve" or "gastric bypass"):ti,ab,kw | 4089 |
| 31 | #29 NOT #30 | 4301 to 4260 trials |

CINAHL (EBSCO)

| 1 | (MH "Animals+") | 105369 |
| --- | --- | --- |
| 2 | (MM "Human") | 177 |
| 3 | S1 not S2 | 105362 |
| 4 | (MH "Randomized Controlled Trials") OR (MH "Intervention Trials") | 142102 |
| 5 | (MH "Clinical Trials") | 187859 |
| 6 | TI ( (randomis* or randomiz* or randomly or trial or multicenter or "multi center" or multicentre or "multi centre" or intervention? or controlled or (control adj group?) or (before adj5 after) or (pre adj5 post) or ((pretest or "pre test") and (posttest or "post test")) or quasiexperiment* or quasi experiment* or evaluat* or "repeated measur*") ) OR AB ( (randomis* or randomiz* or randomly or trial or multicenter or "multi center" or multicentre or "multi centre" or intervention? or controlled or (control adj group?) or (before adj5 after) or (pre adj5 post) or ((pretest or "pre test") and (posttest or "post test")) or quasiexperiment* or quasi experiment* or evaluat* or "repeated measur*") ) | 1721816 |
| 7 | TI ( systematic review or literature review ) OR AB ( systematic review or literature review ) | 236209 |
| 8 | S4 OR S5 OR S6 | 1801063 |
| 9 | S8 NOT S7 | 1686174 |
| 10 | TI ( (("5" or five) N3 ("weight loss" or "weight reduc*" or "weight decreas*" or "weight chang*")) ) OR AB ( (("5" or five) N3 ("weight loss" or "weight reduc*" or "weight decreas*" or "weight chang*")) ) | 445 |
| 11 | TI ( (("weight loss*" N8 stratif*) or ("weight reduc*" N8 stratif*) or ("weight decreas*" N8 stratif*) or ("weight chang*" N8 stratif*)) ) OR AB ( (("weight loss*" N8 stratif*) or ("weight reduc*" N8 stratif*) or ("weight decreas*" N8 stratif*) or ("weight chang*" N8 stratif*)) ) | 85 |
| 12 | TI ( (("5" or five) N3 (decrease or reduc* or loss) N3 weight) ) OR AB ( (("5" or five) N3 (decrease or reduc* or loss) N3 weight) ) | 382 |
| 13 | TI ( (("5" or five or four or three or two or one or "4" or "3" or "2" or "1") N2 (decrease or reduc* or loss) N3 weight) ) OR AB ( (("5" or five or four or three or two or one or "4" or "3" or "2" or "1") N2 (decrease or reduc* or loss) N3 weight) ) | 2094 |
| 14 | TI ( ((four or three or two or one or "4" or "3" or "2" or "1") N2 ("weight loss" or "weight reduc*" or "weight decreas*" or "weight chang*")) ) OR AB ( ((four or three or two or one or "4" or "3" or "2" or "1") N2 ("weight loss" or "weight reduc*" or "weight decreas*" or "weight chang*")) ) | 2209 |
| 15 | TI ( ((satisfactory or moderate or modest or low or less than) N2 ("weight loss" or "weight reduc*" or "weight decreas*")) ) OR AB ( ((satisfactory or moderate or modest or low or less than) N2 ("weight loss" or "weight reduc*" or "weight decreas*")) ) | 1770 |
| 16 | S10 OR S11 OR S12 OR S13 OR S14 OR S15 | 4365 |
| 17 | TI effect? Or impact? or benefit? or improvement? or improve? or relations* or associat* or risk or risks | 1151451 |
| 18 | TI ( ("risk factor*" or (risk adj predict*) or correlation* or biomarker* or causality or associate* or "predisposing factors" or "enabling factors" or "precipitating factors" or "reinforcing factors" or "patient reported outcome*" or prognosis* or prognostic* or mortality) ) OR AB ( ("risk factor*" or (risk adj predict*) or correlation* or biomarker* or causality or associate* or "predisposing factors" or "enabling factors" or "precipitating factors" or "reinforcing factors" or "patient reported outcome*" or prognosis* or prognostic* or mortality) ) | 1511946 |
| 19 | S17 OR S18 | 2185732 |
| 20 | S9 AND S16 AND S19 | 1485 |
| 21 | S20 NOT S3 | 1478 |
| 22 | TI ("bariatric surgery" or "gastric sleeve" or "gastric bypass") | 6243 |
| 23 | S21 NOT S22 | 1411 |
| 24 | S23 **Limiters** - Age Groups: All Adult | 856 |

Applied Social Sciences Index and Abstracts (ProQuest)

| 1 | mainsubject.Exact("animal") NOT mainsubject.Exact("humans") |  |
| --- | --- | --- |
| 2 | mainsubject.Exact("children" OR "adolescent" OR "child" OR "infant") NOT mainsubject.Exact("adult") |  |
| 3 | TI,AB((randomis* OR randomiz* OR randomly OR trial OR multicenter OR "multi center" OR multicentre OR "multi centre" OR intervention? OR controlled OR (control adj group?) OR (before adj5 after) OR ("pre" adj5 post) OR ((pretest OR "pre test") AND (posttest OR "post test")) OR quasiexperiment* OR quasi experiment* OR evaluat* OR "repeated measur*")) NOT TI(systematic review OR literature review) | 295,102 |
| 4 | TI,AB(("5" or five) NEAR/3 ("weight loss" or "weight reduc*" or "weight decreas*" or "weight chang*")) OR TI,AB(("weight loss*" NEAR/8 stratif*) OR ("weight reduc*" NEAR/8 stratif*) OR ("weight decreas*" NEAR/8 stratif*) OR ("weight chang*" NEAR/8 stratif*)) OR TI,AB(("5" or five) NEAR/3 (decrease or reduc* or loss) NEAR/3 weight) OR TI,AB(("5" or five or four or three or two or one or "4" or "3" or "2" or "1") NEAR/2 (decrease or reduc* or loss) NEAR/3 weight) OR TI,AB((four or three or two or one or "4" or "3" or "2" or "1") NEAR/2 ("weight loss" or ("weight reduced" OR "weight reducing" OR "weight reduction" OR "weight reductions") or ("weight decreased" OR "weight decreases") or ("weight change" OR "weight changes"))) OR TI,AB(((satisfactory or moderate or modest or low or "less than") NEAR/2 ("weight loss" or ("weight reduced" OR "weight reducing" OR "weight reduction" OR "weight reductions") or ("weight decreased" OR "weight decreases")))) | 667 |
| 5 | TI,AB(("risk factor" OR "risk factors") or (risk adj predict*) or correlation* or biomarker* or causality or associate* or "predisposing factors" or "enabling factors" or "precipitating factors" or "reinforcing factors" or "patient reported outcome*" or prognosis* or prognostic* or mortality) |  |
| 6 | TI,AB(("risk factor" OR "risk factors") or (risk adj predict*) or correlation* or biomarker* or causality or associate* or "predisposing factors" or "enabling factors" or "precipitating factors" or "reinforcing factors" or "patient reported outcome*" or prognosis* or prognostic* or mortality) OR TI(effect? Or impact? or benefit? or improvement? or improve? or relations* or associat* or risk or risks) | 369,516 |
| 7 | ((TI,AB((randomis* OR randomiz* OR randomly OR trial OR multicenter OR "multi center" OR multicentre OR "multi centre" OR intervention? OR controlled OR (control adj group?) OR (before adj5 after) OR ("pre" adj5 post) OR ((pretest OR "pre test") AND (posttest OR "post test")) OR quasiexperiment* OR quasi experiment* OR evaluat* OR "repeated measur*")) NOT TI(systematic review OR literature review)) AND (TI,AB(("5" or five) NEAR/3 ("weight loss" or "weight reduc*" or "weight decreas*" or "weight chang*")) OR TI,AB(("weight loss*" NEAR/8 stratif*) OR ("weight reduc*" NEAR/8 stratif*) OR ("weight decreas*" NEAR/8 stratif*) OR ("weight chang*" NEAR/8 stratif*)) OR TI,AB(("5" or five) NEAR/3 (decrease or reduc* or loss) NEAR/3 weight) OR TI,AB(("5" or five or four or three or two or one or "4" or "3" or "2" or "1") NEAR/2 (decrease or reduc* or loss) NEAR/3 weight) OR TI,AB((four or three or two or one or "4" or "3" or "2" or "1") NEAR/2 ("weight loss" or ("weight reduced" OR "weight reducing" OR "weight reduction" OR "weight reductions") or ("weight decreased" OR "weight decreases") or ("weight change" OR "weight changes"))) OR TI,AB(((satisfactory or moderate or modest or low or "less than") NEAR/2 ("weight loss" or ("weight reduced" OR "weight reducing" OR "weight reduction" OR "weight reductions") or ("weight decreased" OR "weight decreases"))))) AND (TI,AB(("risk factor" OR "risk factors") or (risk adj predict*) or correlation* or biomarker* or causality or associate* or "predisposing factors" or "enabling factors" or "precipitating factors" or "reinforcing factors" or "patient reported outcome*" or prognosis* or prognostic* or mortality) OR TI(effect? Or impact? or benefit? or improvement? or improve? or relations* or associat* or risk or risks))) NOT ((mainsubject.Exact("animal") NOT mainsubject.Exact("humans")) OR (mainsubject.Exact("children" OR "adolescent" OR "child" OR "infant") NOT mainsubject.Exact("adult")) OR TI("bariatric surgery" or "gastric sleeve" or "gastric bypass")) | 198 |

Web of Science - social science and emerging sources

| 1 | TS=((randomis* or randomiz* or randomly or trial or multicenter or "multi center" or multicentre or "multi centre" or intervention? or controlled or (control adj group?) or (before NEAR/5 after) or (pre NEAR/5 post) or ((pretest or "pre test") and (posttest or "post test")) or quasiexperiment* or quasi experiment* or evaluat* or "repeated measur*")) | 2464349 |
| --- | --- | --- |
| 2 | TS=((5 or five) NEAR/3("weight loss" or "weight reduc*" or "weight decreas*" or "weight chang*")) | 1205 |
| 3 | TS=(("weight loss*" NEAR/8 stratif*) or ("weight reduc*" NEAR/8 stratif*) or ("weight decreas*" NEAR/8 stratif*) or ("weight chang*" NEAR/8 stratif*)) | 46 |
| 4 | TS=((("5" or five) NEAR/3 (decrease or reduc* or loss) NEAR/3 weight)) | 1233 |
| 5 | TS=(((("5" or five or four or three or two or one or "4" or "3" or "2" or "1") NEAR/2 (decrease or reduc* or loss)) NEAR/1 weight)) | 3203 |
| 6 | TS=((four or three or two or one or "4" or "3" or "2" or "1") NEAR/1 ("weight loss" or "weight reduc*" or "weight decreas*" or "weight chang*")) | 2727 |
| 7 | TS=(("satisfactory" or "moderate" or "modest" or "low" or "less than") NEAR/2 ("weight loss" or "weight reduc*" or "weight decreas*")) | 713 |
| 8 | TI=(impact? or benefit? or improvement? or improve? or relations* or associat* or risk or risks) | 762382 |
| 9 | TS=(("risk factor*" or (risk adj predict*) or correlation* or biomarker* or causality or associate* or "predisposing factors" or "enabling factors" or "precipitating factors" or "reinforcing factors" or "patient reported outcome*" or prognosis* or prognostic* or mortality)) | 1694086 |
| 10 | #2 OR #3 OR #4 OR #5 OR #6 OR #7 | 4511 |
| 11 | #8 OR #9 | 2118064 |
| 12 | #1 AND #10 AND #11 | 1664 |
| 13 | TI=("bariatric surgery" or "gastric sleeve" or "gastric bypass" or "systematic review" or "literature review") | 105253 |
| 14 | TS=(animal) | 116411 |
| 15 | #13 OR #14 | 220496 |
| 16 | #12 NOT #15 | 1481 |

**Table S2** – CASP bias assessment

| **Author (Year)** | **1** | **2** | **3** | **4a** | **4b** | **4c** | **5** | **6** | **7** | **8** | **9** |
| --- | --- | --- | --- | --- | --- | --- | --- | --- | --- | --- | --- |
| Abbenhardt (2013) |  |  |  |  |  |  |  |  |  |  |  |
| Ahmand (2020) |  |  |  |  |  |  |  |  |  |  |  |
| Ahn (2018) |  |  |  |  |  |  |  |  |  |  |  |
| Alfaris (2015) |  |  |  |  |  |  |  |  |  |  |  |
| Aller (2015) |  |  |  |  |  |  |  |  |  |  |  |
| Ashley (2001) |  |  |  |  |  |  |  |  |  |  |  |
| Bays (2017) |  |  |  |  |  |  |  |  |  |  |  |
| Campbell (2012) |  |  |  |  |  |  |  |  |  |  |  |
| Chang (2010) |  |  |  |  |  |  |  |  |  |  |  |
| Chang (2019) |  |  |  |  |  |  |  |  |  |  |  |
| Christian (2011) |  |  |  |  |  |  |  |  |  |  |  |
| D'Alonzo (2021) |  |  |  |  |  |  |  |  |  |  |  |
| Davidson (2013) |  |  |  |  |  |  |  |  |  |  |  |
| Del Bel (2012) |  |  |  |  |  |  |  |  |  |  |  |
| Dittus (2018) |  |  |  |  |  |  |  |  |  |  |  |
| Dong (2021) |  |  |  |  |  |  |  |  |  |  |  |
| Duggan (2015) |  |  |  |  |  |  |  |  |  |  |  |
| Duggan (2016) |  |  |  |  |  |  |  |  |  |  |  |
| Falchi (2014) |  |  |  |  |  |  |  |  |  |  |  |
| Georgoulis (2022) |  |  |  |  |  |  |  |  |  |  |  |
| Gomez-Huelgas (2019) |  |  |  |  |  |  |  |  |  |  |  |
| Grandi (2019) |  |  |  |  |  |  |  |  |  |  |  |
| Habermann (2015) |  |  |  |  |  |  |  |  |  |  |  |
| Harrigan (2016) |  |  |  |  |  |  |  |  |  |  |  |
| Höchsmann, (2021) |  |  |  |  |  |  |  |  |  |  |  |
| Imayama (2012) |  |  |  |  |  |  |  |  |  |  |  |
| Johnson (2011) |  |  |  |  |  |  |  |  |  |  |  |
| Jouneau (2020) |  |  |  |  |  |  |  |  |  |  |  |
| Kaholokula (2013) |  |  |  |  |  |  |  |  |  |  |  |
| Kiddy (1992) |  |  |  |  |  |  |  |  |  |  |  |
| Kolehmainen (2008) |  |  |  |  |  |  |  |  |  |  |  |
| Kolotkin (2009) |  |  |  |  |  |  |  |  |  |  |  |
| Konerman (2019) |  |  |  |  |  |  |  |  |  |  |  |
| Kosiborod (2022) |  |  |  |  |  |  |  |  |  |  |  |
| Lang (2011) |  |  |  |  |  |  |  |  |  |  |  |
| Magkos (2017) |  |  |  |  |  |  |  |  |  |  |  |
| Maruthur (2013) |  |  |  |  |  |  |  |  |  |  |  |
| Mason (2015) |  |  |  |  |  |  |  |  |  |  |  |
| Messier (2009) |  |  |  |  |  |  |  |  |  |  |  |
| Miazgowski (2021) |  |  |  |  |  |  |  |  |  |  |  |
| Muls (2001) |  |  |  |  |  |  |  |  |  |  |  |
| Muramoto (2014) |  |  |  |  |  |  |  |  |  |  |  |
| Nadinskaia (2021) |  |  |  |  |  |  |  |  |  |  |  |
| Nagahara (2021) |  |  |  |  |  |  |  |  |  |  |  |
| Patrick – study 2 |  |  |  |  |  |  |  |  |  |  |  |
| Perreault (2008) |  |  |  |  |  |  |  |  |  |  |  |
| Poppitt (2002) |  |  |  |  |  |  |  |  |  |  |  |
| Rock (2013) |  |  |  |  |  |  |  |  |  |  |  |
| Rusu (2013) |  |  |  |  |  |  |  |  |  |  |  |
| Scott (2012) |  |  |  |  |  |  |  |  |  |  |  |
| Sharma (2009) |  |  |  |  |  |  |  |  |  |  |  |
| Sheng (2022) |  |  |  |  |  |  |  |  |  |  |  |
| Shirai (2013) |  |  |  |  |  |  |  |  |  |  |  |
| Smith (2011) |  |  |  |  |  |  |  |  |  |  |  |
| Spurny (2020) |  |  |  |  |  |  |  |  |  |  |  |
| St. George (2009) |  |  |  |  |  |  |  |  |  |  |  |
| Swift (2016) |  |  |  |  |  |  |  |  |  |  |  |
| Swift (2018) |  |  |  |  |  |  |  |  |  |  |  |
| Thibault (2015) |  |  |  |  |  |  |  |  |  |  |  |
| Tseng (2002) |  |  |  |  |  |  |  |  |  |  |  |
| Vasiljevic (2012) |  |  |  |  |  |  |  |  |  |  |  |
| Vetter (2013) |  |  |  |  |  |  |  |  |  |  |  |
| Wing (2010) |  |  |  |  |  |  |  |  |  |  |  |
| Wing (2011) |  |  |  |  |  |  |  |  |  |  |  |
| Wu (2009) |  |  |  |  |  |  |  |  |  |  |  |

| **Author (Year)** | **1** | **2** | **3** | **4** | **5a** | **5b** | **6a** | **6b** | **7** | **8** | **9** | **10** | **11** | **12** |
| --- | --- | --- | --- | --- | --- | --- | --- | --- | --- | --- | --- | --- | --- | --- |
| Annessi (2023) |  |  |  |  |  |  |  |  | NA |  |  |  |  | NA |
| Rintamaki (2021) |  |  |  |  |  |  |  |  | NA |  |  |  |  | NA |
| Strelitz (2019) |  |  |  |  |  |  |  |  | NA |  |  |  |  | NA |

**Table S3 –** List of all the specific health markers and indices identified within the included studies

| **Cardiometabolic** | Diastolic blood pressure, systolic blood pressure, blood pressure, mean arterial pressure, triglycerides, total cholesterol, high-density lipoprotein cholesterol, low-density lipoprotein cholesterol, non-high-density lipoprotein cholesterol, mean non- high-density lipoprotein cholesterol, total high-density lipoprotein cholesterol ratio, small low-density lipoprotein cholesterol, large high-density lipoprotein cholesterol, low-density lipoprotein cholesterol particles, total cholesterol/high-density lipoprotein cholesterol ratio, protein intake, P wave dispersion, and cardiovascular disease incidence |
| --- | --- |
| **Metabolic** | Adiponectin, leptin, fasted state leptin, fasting plasma glucose, fasting insulin, fasting serum insulin, fasting glucose, 2-h plasma glucose concentration, frequently sampled intravenous glucose tolerance test, hemoglobin A1C, homeostatic model assessment for insulin resistance, homeostasis model assessment of β-cell function, matsuda index, 30-min insulin test, glucose area under the curve, sum of insulin concentrations, carbohydrate‐to‐insulin ratio, insulin sensitivity, glucose effectiveness, acute insulin response, adipocyte cell size, C-reactive protein, insulin-like growth factor 1, diabetes risk, diabetes incidence, fasting total serum ghrelin, irisin, fibrosis, controlled attenuation parameter, microbiome, DNA repair capacity, radiation sensitivity, metabolic syndrome severity Z-score and metabolic syndrome components, metabolic syndrome incidence and escape rate, vaspin, C-peptide, fibrinogen, resting energy expenditure, estrone, estradiol, total testosterone, sex hormone–binding globulin, free estradiol, free testosterone, Urinary 8-isoprostanes, soluble NOX2-derived peptide, vitamin E, vitamin E/cholesterol ratio, and bone marrow fat content |
| **Anthropometric** | Waist circumference, hip circumference, abdominal circumference, percent body fat, fat mass, fat free mass, trunk fat mass, visceral fat area, visceral fat mass, visceral adipose tissue, body fat composition, subcutaneous fast mass, lean body mass, android fat, and gynoid fat |
| **Quality of life** | Sleep duration and quality, sleep mood (PHQ-8 test), sleep disturbance, apnoea-hypopnea index, non-rapid eye movement apnea-hypopnea index, rapid eye movement apnea-hypopnea index, hypopnea index, oxygen saturation in the arterial blood, asthma quality of life questionnaire domains, asthma control score, impact of weight on quality of life-lite scores, EQ-5D scores, SF-36 scores, OWLQOL score, WSRM bother scores, physical functioning, pain, social functioning, role functioning, mental health, health perceptions, self-efficacy, perceived risk for heart disease and diabetes, impact of weight on QOL-Lite, urinary incontinence per week (total, stress and urge), 24-hour involuntary urine loss, and satisfaction with changes with incontinence |
| **Inflammatory biomarkers** | Tumor necrosis factor α, high-sensitivity C-reactive protein, interleukin-1 beta, interleukin-6, interleukin-8, interleukin-10, inflammatory biomarker score, oxidized low-density lipoprotein, fluorescent oxidation products, F2-isoprostanes, resistin, airway inflammation, interleukins, vascular endothelial growth factor, vitamin D, serum amyloid A, leukocytes, and neutrophils |
| **Renal and hepatic** | Angiotensinogen, malondialdehyde, gamma-glutamyl transferase, monocyte chemoat-tractant protein-1, podocalyxin, 24-hr Una/Cr, estimated glomerular filtration rate, albuminuria, albumin, bilirubin, fatty liver index, non-alcoholic fatty liver disease fibrosis score, uric acid, alanine transaminase, aspartate aminotransferase, gamma-glutamyl transpeptidase, fatty liver index, aspartate transaminase/alanine transaminase ratio, Forns fibrosis index and AST to platelet ratio index |
| **Psychosocial and behavioral** | Physical activity- and eating-related self-regulation, self-efficacy, mood, emotional eating, physical function, sexual function, endocrine symptoms, pain interference, fatigue, depression, anxiety, sleep disturbance, total body esteem, appearance, attribution, self-esteem, stress, dietary restraint, disinhibition, hunger, physical activity MET hrs/week, exercise fatigue level, 6-min walk test, exercise frequency, fat in diet, PHQ-9 depression score, Brief Symptom Rating Scale, Bulimic Investigatory Test, Edinburgh, role-physical, bodily pain, general health, vitality, social functioning, role-emotional, mental health, physical component summary scores, and mental component summary scores |
| **Pulmonary function** | Functional residual capacity, expiratory reserve volume, residual volume, inspiratory capacity, total lung capacity, forced expiratory volume in one-second, forced vital capacity, forced expiratory volume in one-second/forced vital capacity ratio, asthma control questionnaire, asthma health-related quality of life, St. George’s Respiratory Questionnaire |
| **Muscle strength** | Quadriceps muscle strength and endurance |

**Table S4** – Sensitivity tables for time follow up and change in health measures

|  | **Change in health measures** | | | |  |
| --- | --- | --- | --- | --- | --- |
| **Follow-up period** | Improvement | Worsening | Mixed | No change | **Total** |
| Greater than 6 months | 47 (60%) | 3 (4%) | 18 (23%) | 10 (13%) | 78 |
| Less than or equal to 6 months | 37 (63%) | 4 (7%) | 10 (17%) | 8 (13%) | 59 |
| **Total** | 84 | 7 | 28 | 18 | 137 |

|  | **Change in health measures** | | | |  |
| --- | --- | --- | --- | --- | --- |
| **Follow-up period** | Improvement | Worsening | Mixed | No change | **Total** |
| Greater than 12 months | 19 (59%) | 2 (6%) | 7 (22%) | 4 (13%) | 32 |
| Less than or equal to 12 months | 65 (62%) | 5 (5%) | 21 (20%) | 14 (13%) | 105 |
| **Total** | 84 | 7 | 28 | 18 | 137 |

**Table S5** – Detailed description of included studies

| **Author (year), country,**  **study design** | **Participant characteristics (age, year; BMI, kg/m^2^; inclusion criteria for BMI and co-morbidities)** | **Intervention characteristics** | **Duration** | **Follow up** | **Health outcomes** | **Key findings** |
| --- | --- | --- | --- | --- | --- | --- |
| **Abbenhardt** (2013)  USA  RCT | **Mean age ± SD:** 58.0 ± 5.0  **Baseline mean BMI:**  Group 1: 30.7 ± 3.7  Group 2: 31.0 ± 3.9  Group 3: 31.0 ± 4.3  Group 4: 30.7 ± 3.9  **Sample size:** 439  **Inclusion criteria:**  **BMI**: Overweight (BMI 25-29.9 kg/m^2^) and higher  **Co-morbidities**: NA | **Lifestyle interventions:**   - Group 1: Aerobic exercise intervention - Group 2: Dietary weight loss intervention - Group 3: Diet + exercise intervention - Group 4: Control | 12 months | Intervention end | **Metabolic**  APN and leptin | Results were stratified into <5% weight loss, 5-10% weight loss, and ≥ 10% weight loss. For the <5% group there was a significant decrease in leptin for group 1. Adiponectin decreased in all groups; leptin increased in group 3, but none were significant. |
| **Ahmad** (2020)  Malaysia  Quasi-experimental | **Mean age:**  Group 1: 42.41 ± 8.59  Group 2: 41.66 ± 8.65  **Baseline mean BMI (>2–<5% loss):**  Group 1: 30.12 ± 3.86  Group 2: 32.23 ± 4.59  **Sample size:** 243  **Inclusion criteria:**  **BMI**: Overweight (BMI 25-29.9 kg/m^2^) and higher  **Co-morbidities**: NA | **Lifestyle intervention:**   - Group 1: Monthly 1-hour individual diet counselling and group moderate physical activity sessions - Group 2: Control, received general women’s health seminars during follow-up | 12 months | 6 months & intervention end | **Anthropometric**  WC  **Cardiovascular**  TG, TC, HDL-C, LDL-C  **Metabolic**  FPG, fasting insulin, HOMA-IR, and APN  **Inflammatory biomarkers**  TNF -α, and hs-CRP | Results were stratified into 2% gain, ±2% maintain, >2 to <5% loss, and 5 to 20% loss (for both intervention and control). For the participants in the >2 to <5% weight loss group, there was a significant decrease for WC (intervention and control at 0-6 and 6-12 months), FPG (control at 6-12 months), HDL-C (intervention and control at 6-12 months), LDL-C (intervention at 6-12 months), HOMA-IR (intervention at 0-6 months), hs-CRP (intervention at 0-6 months), and TNF-α (intervention at 6-12 months). |
| **Ahn** (2018)  South Korea  Open label, RCT | **Mean age:** 50.3 ± 13.0  **Baseline mean BMI:** 25.4 ± 3.8  **Sample size:** 277  **Inclusion criteria:**  **BMI**: NA  **Co-morbidities:** Metabolic syndrome and chronic kidney disease | **Lifestyle and pharmacological interventions**   - Group 1: Daily fixed dose of Olmesartan medoxomil (40 mg) for 16 weeks + intensive education for a low-salt diet from week 8-16 (dietary consultant and telephone feedback) - Group 2: Daily fixed dose of Olmesartan medoxomil (40 mg) for 16 weeks + conventional education for a low-salt diet from week 8-16 (control) | 16 weeks | Intervention end | **Cardiovascular**  SBP, TC, and protein intake  **Metabolic**  APN  **Renal and hepatic**  AGT, MDA, MCP-1, podocalyxin, 24-hr Una/Cr, eGFR, and albuminuria | Results were stratified into weight increased ≥ 0.0%, decreased 1.5-0.5%, and decreased ≥ 1.5% (the mean difference in weight was -2.41 +/- 1.45). In the decreased 1.5-0.5%, and ≥ 1.5% groups there was a significant decrease in 24-hr Una/Cr, podocalyxin, and albuminuria levels, compared to the group that gained weight. There were non-significant decreases in SBP, TC, eGFR, APN, AGT, MCP-1, MDA, and increases in protein intake. |
| **Alfaris** (2015)  USA  RCT | **Mean age:** 51.5 ± 11.5  **Baseline mean BMI:** 38.5 ± 4.7  **Sample size:** 390  **Inclusion criteria:**  **BMI**: Obesity class 1 (BMI 30-34.9 kg/m^2^) and higher **Co-morbidities:** Metabolic syndrome | **Lifestyle interventions**   - Group 1: Brief lifestyle counseling. - Group 2: Enhanced brief lifestyle counseling. - Group 3: Usual care (control)   All participants received the same diet and physical activity goals but received different amounts of behavioral support to reach these targets. | 24 months | 6 months & intervention end | **Quality of life**  Sleep duration and quality (PSQI), and mood (PHQ-8) | Results were stratified into weight decreased <5% and ≥5%. For the <5% group, at 6 months there was an increase of in the number of participants who reported suboptimal sleep duration at baseline (19.5%); sleep duration increased, sleep quality and changes in mood improved (minimal at 6 months, greater at 24 months). |
| **Aller** (2015)  Spain  RCT | **Mean age:** 47.4 ± 11.2  **Baseline mean BMI:**  Group 1: 36.8 ± 7.9  Group 2: 35 ± 7.4  **Sample size:** 36  **Inclusion criteria:**  **BMI**: NA  **Co-morbidities:** Non-alcoholic fatty liver disease | **Lifestyle and pharmacological interventions:**   - Group 1: Two tablets of silymarin per day (540.3 mg) + vitamin E (36 mg) + exercise + hypocaloric diet - Group 2: Hypocaloric diet only | 3 months | Intervention end | **Renal and hepatic**  GGT, FLI, NAFLD-fibrosis score | Results were stratified into weight decreased <5% and ≥5%. The participants who lost <5% body weight in group 1 had a significant decrease in GGT levels, FLI and NAFLD-FS. No changes in participants who lost less than 5% body weight in group 2. |
| **Annesi** (2023)    USA  Non-randomised intervention study | **Mean age:** 47.4 ± 8.6  **Baseline mean BMI:** 34.7 ± 3.2  **Sample size:** 128  **Inclusion criteria:**  **BMI**: Obesity class 1 (BMI 30-34.9 kg/m^2^) and higher  **Co-morbidities:** NA | **Lifestyle intervention:**   - Physical activity support sessions and group-based sessions of 50 minutes, each focused on controlling eating and self-regulation | 14 months | 6 months, intervention end & 24 months | **Psychosocial and behavioral**  Physical activity- and eating-related self-regulation and self-efficacy, mood, and emotional eating | Participants were stratified into weight loss groups, minimal effect group (lost less than 5% by month 6); loss/regain group (lost 5% or more and 2% or more was regained during 6 to 24 months); loss/loss group (lost 5% or more and less than 2% regained/further weight loss during 6 to 24 months). For the minimal effect group, all outcomes improved from baseline to 6 months (small - large effects). Later outcomes were not reported for this group. |
| **Ashley** (2001)  USA  RCT | **Mean age:** 41.1 ± 4.7 (n = 74 (excluding non-completers)  **Baseline mean BMI:** 30 ± 3  **Sample size:** 113  **Inclusion criteria:**  **BMI**: Overweight (BMI 25-29.9 kg/m^2^) and higher  **Co-morbidities:** NA | **Lifestyle intervention:**   - Group 1: Traditional dietitian-led intervention group - Group 2: Traditional dietitian-led intervention group incorporating meal replacements - Group 3: Primary care office intervention incorporating meal replacements with individual physician and nurse visits | 12 months | Intervention end | **Anthropometric**  WC  **Cardiovascular**  SBP, DBP, TC, TG, HDL-C, LDL-C  **Metabolic**  Resting energy expenditure, and fasting glucose/insulin | Results were stratified into weight decreased <5%, 5-10%, and ≥10%. For the <5% weight loss group, glucose significantly increased; no other outcomes were significantly different. |
| **Bays** (2017) - Study 1 SCALE Obesity  USA + Canada  Secondary analysis of an RCT | **Mean age (SD):** 45.1 (12.0)  **Baseline mean BMI (SD):**  38.3 (6.4)  **Sample size:** 3731  **Inclusion criteria:**  **BMI**: Overweight (BMI 25-29.9 kg/m^2^) and higher  **Co-morbidities:** NA | **Pharmacological and lifestyle interventions:**   - Group 1: Daily Liraglutide 3.0 mg and lifestyle intervention (reduced calorie diet and increased physical activity) - Group 2: Placebo and lifestyle intervention | 56 weeks | Intervention end | **Cardiovascular**  SBP | Results were stratified by weight gain, 0-4.9%, 5-9.9%, 10-14.9%, and ≥15%.  For the participants who lost 0-4.9% weight in the liraglutide group SBP decreased (significance not tested). |
| **Bays** (2017) - Study 2 SCALE Diabetes  USA + Canada  Secondary analysis of an RCT | **Mean age (SD):** 54.9 (10.5)  **Baseline mean BMI (SD):**  37.2 (6.7)  **Sample size:** 635  **Inclusion criteria:**  **BMI**: Overweight (BMI 25-29.9 kg/m^2^) and higher  **Co-morbidities:** Type 2 Diabetes | **Pharmacological and lifestyle interventions:**   - Group 1: Daily Liraglutide 3.0 mg and lifestyle intervention (reduced calorie diet and increased physical activity) - Group 2: Placebo and lifestyle intervention | 56 weeks | Intervention end | **Metabolic**  HbA1c and FPG | Results were stratified by weight gain, 0-4.9%, 5-9.9%, 10-14.9%, and ≥15%. For the participants who lost 0-4.9% weight in the liraglutide group FPG and HbA1C all decreased; in the placebo group all but FPG decreased (significance not tested). |
| **Bays** (2017) - Study 3 SCALE Sleep Apnea  USA + Canada  Secondary analysis of an RCT | **Mean age (SD):** 48.5 (9.7)  **Baseline mean BMI (SD):**  39.1 (6.9)  **Sample size:** 359  **Inclusion criteria:**  **BMI**: Overweight (BMI 25-29.9 kg/m^2^) and higher  **Co-morbidities:** Obstructive sleep apnea | **Pharmacological and lifestyle interventions:**   - Group 1: Daily Liraglutide 3.0 mg and lifestyle intervention (reduced calorie diet and increased physical activity) - Group 2: Placebo and lifestyle intervention | 32 weeks | Intervention end | **Quality of life**  AHI | Results were stratified by weight gain, 0-4.9%, 5-9.9%, 10-14.9%, and ≥15%. For the participants who lost 0-4.9% weight in the liraglutide group AHI decreased (significance not tested). |
| **Campbell** (2012)  USA  RCT | **Mean age (SD):** 58 (5.0)  **Baseline mean BMI (SD):**  30.9 (4.0)  **Sample size:** 421  **Inclusion criteria:**  **BMI**: Overweight (BMI 25-29.9 kg/m^2^) and higher  **Co-morbidities:** NA | **Lifestyle interventions:**   - Group 1: Reduced-calorie diet - Group 2: Moderate-vigorous aerobic exercise - Group 3: Diet + exercise - Group 4: Control | 12 months | Intervention end | **Metabolic**  Estrone, estradiol, total testosterone, sex hormone–binding globulin, free estradiol, and free testosterone | Results were stratified by weight loss <5%; 5-10% and ≥10%. For the participants who lost <5% weight in group 1 and 3, there were improvements in all but sex hormone–binding globulin. The significance in the difference from baseline was not tested. |
| **Chang** (2010)  South Korea  Longitudinal clinical intervention study | **Mean age:** 38.2 ± 1.3  **Baseline mean BMI:** 31.9 ± 0.3  **Sample size:** 63  **Inclusion criteria:**  **BMI**: Overweight (BMI 25-29.9 kg/m^2^) and higher  **Co-morbidities:** Metabolic syndrome | **Lifestyle and pharmacological intervention:**   - Reduce their daily energy intake by 500 kcal, exercise regularly (40–50 min/day, 4–5 days/week) and monthly consultations with a dietitian and a physician - Orlistat (120mg) three times daily. | 12 weeks | Intervention end | **Anthropometric**  WC and hip circumference  **Cardiovascular**  TC, TG, HDL-C, LDL-C  **Metabolic**  Fasting glucose, HbA1c, fasting insulin, HOMA-IR, and vaspin | Results were stratified into weight decrease <2% and ≥2%. In the <2% group, significant improvements were seen for waist and hip circumference; and significant worsening for TG, fasting glucose and insulin, HOMA-IR and Vaspin. |
| **Chang** (2019)  USA  RCT | **Mean age (SD):** 28.5 (5.03)  **Baseline mean BMI (SD):**  32.04 (4.29)  **Sample size:** 569  **Inclusion criteria:**  **BMI**: Overweight (BMI 25-29.9 kg/m^2^) and higher  **Co-morbidities:** NA | **Lifestyle intervention:**   - Group 1: 4-month intervention with videos and peer-education on sleep, stress, healthy eating and physical activity - Group 2: Control | 4 months | Baseline (T1), 4-month (T2), 7-month (T3) | **Quality of life**  Sleep duration and quality, and sleep disturbance | Results were stratified by weight loss <5%; and ≥5%. In the participants who lost <5% weight the sleep duration, sleep quality and sleep disturbance all improved between T1 and both T2 and T3 (significance not tested). |
| **Christian** (2011)  USA  Prospective cluster RCT | **Mean age:**  Group 1: 49.2 ± 13.04  Group 2: 50.0 ± 11.79  **Baseline mean BMI:**  Group 1: 34.7 ± 7.39  Group 2: 33.8 ± 7.34  **Sample size:** 279  **Inclusion criteria:**  **BMI**: Overweight (BMI 25-29.9 kg/m^2^) and higher  **Co-morbidities:** Metabolic syndrome | **Lifestyle interventions:**   - Group 1: Tailored self-management goals for weight loss, nutrition, and physical activity, reviewed at clinic visits. - Group 2: Usual care – given information booklet on health education | 12 months | Intervention end | **Anthropometric**  WC  **Cardiovascular**  TC, TG, HDL-C, LDL-C, SBP, DBP  **Metabolic**  Fasting glucose and fasting insulin  **Psychosocial and behavioral**  Physical activity MET hrs/week | Results were stratified into weight decreased <5% and >5%. For the <5% group, there were no significant changes, but non-significant improvements seen for SBP, DBP, TC, HDL-C, TG, LDL-C. No difference by SES/ethnicity. |
| **D'Alonzo** (2021)  USA  RCT | **Mean age (SD):** 59.9 (8.9)  **Baseline mean BMI (SD):**  33.8 (5.9)  **Sample size:** 206  **Inclusion criteria:**  **BMI**: Overweight (BMI 25-29.9 kg/m^2^) and higher  **Co-morbidities:** NA | **Lifestyle intervention:**   - Group 1: exercise - Group 2: caloric restriction - Group 3: exercise + caloric restriction - Group 4: control | 12 months | Intervention end | **Metabolic**  Insulin, C-peptide, glucose, HOMA2-IR, HOMA2-β | Results were stratified by weight loss 0–5%; ≥5–10; and ≥10. In the participants who lost 0-5% weight, there were improvements in all metabolic markers except for HOMA2-β (significance not tested). |
| **Davidson** (2013)  USA  RCT | **Mean age (SD):** 51.1 (10.4)  **Baseline mean BMI (SD):**  36.6 (4.5)  **Sample size:** 2,487  **Inclusion criteria:**  **BMI**: Overweight (BMI 25-29.9 kg/m^2^) and higher  **Co-morbidities:** Metabolic syndrome | **Pharmacological and lifestyle interventions:**   - Group 1: PHEN 7.5 mg/TPM ER 46 mg (7.5/46) - Group 2: PHEN 15 mg/TPM ER 92 mg (15/92) - Group 3: Placebo + lifestyle modification | 56 weeks | Intervention end | **Cardiovascular**  TG, SBP, DBP, HDL-C, LDL-C, Non-HDL cholesterol  **Metabolic**  APN and fibrinogen  **Inflammatory biomarkers**  h-CRP | Results were stratified by weight loss <5%; ≥5–<10; ≥10-<15%; and ≥15%. In the participants who lost <5% weight, there were improvements in all assessed cardiovascular, metabolic and inflammatory markers (all were significant except for fibrinogen). |
| **Del Ben** (2012)  Italy  Non-randomised intervention | **Age range:** 40-80 (mean age NR)  **Baseline mean body weight (kg) (<5% weight loss):** 98.7 ± 18.2 (BMI NR)  **Sample size:** 172  **Inclusion criteria:**  **BMI**: NA  **Co-morbidities:** Metabolic syndrome | **Lifestyle intervention:**   - Moderately calorie-restricted diet – a balanced low-calorie (600 calories/day negative energy balance), low-fat, high-carbohydrate diet. | 6 months | Intervention end | **Cardiovascular**  TC  **Metabolic**  Urinary 8-isoprostanes, sNOX2-dp, APN, vitamin E, and vitamin E/cholesterol ratio | Results were stratified into weight decreased <5% and ≥5%. In the <5% group, there were no significant improvements. The metabolic outcomes had non-significant improvements. |
| **Dittus** (2018)  USA  RCT | **Mean age (<5% weight loss):** 54.25 ± 4.78  **Baseline mean BMI (<5% weight loss):** 34.48 ± 7.71  **Sample size:** 76  **Inclusion criteria:**  **BMI**: Overweight (BMI 25-29.9 kg/m^2^) and higher  **Co-morbidities:** NA | **Lifestyle intervention:**   - Group 1: A behavioral, online weight control program including calorie restriction, physical activity and behavioral modification principles. - Group 2: The above intervention with added resistance training. | 6 months | Intervention end | **Anthropometric**  % body fat, fat mass, fat free mass  **Metabolic**  Fasting glucose, fasting insulin, Matsuda index, 30-min insulin, HOMA-IR, AUC glucose/insulin, HOMA-β, leptin, and APN | Results were stratified into weight decreased <5% and ≥5%. In the <5% group, the within group changes were not tested. Observed changes were a decrease in APN, leptin and fat free mass, but increases in insulin and glucose measures. |
| **Dong** (2021)  USA  Prospective intervention study | **Mean age [IQR]:** 57 [18]  **Baseline median BMI [IQR]:** 34.3 [4.7]  **Sample size:** 80  **Inclusion criteria:**  **BMI**: Overweight (BMI 25-29.9 kg/m^2^) and higher  **Co-morbidities:** NA | **Lifestyle intervention:**   - Macronutrient standardized diet for 16 weeks, including 14 weeks of calorie restriction (500 calorie deficit). | 16 weeks | Intervention end | **Cardiovascular**  TC, HDL-C, LDL-C, TG  **Metabolic**  HbA1c, fibrosis, controlled attenuation parameter, and microbiome | Results were stratified into weight decreased <5% and ≥5%. In the <5% group, the microbiome diversity increased, but was not significant. The other within group changes were not tested. The observed differences were a decrease in TC, HDL, LDL, TG and an increase in the controlled attenuation parameter. |
| **Duggan** (2015)  USA  RCT | **Mean age:** 59.6 ± 5.1  **Baseline mean BMI:** 32.4 ± 5.8  **Sample size:** 218  **Inclusion criteria:**  **BMI**: Overweight (BMI 25-29.9 kg/m^2^) and higher  **Co-morbidities:** Insufficient serum 25 (OH) D concentration | **Lifestyle and pharmacological intervention:**   - Group 1: Weight-loss intervention (diet and exercise) + 2000 IU/day oral vitamin D_3_. - Group 2: Weight-loss intervention (diet and exercise) + daily placebo pill. | 12 months | Intervention end | **Metabolic**  APN and leptin  **Inflammatory biomarkers**  IL1β, IL6 IL8, IL10, TNF-α, and inflammatory biomarker score | Results were stratified into gained/no weight-loss, <5%, 5-10%, and ≥10%. In the <5% weight loss group the within group changes were not tested. In both groups the following outcomes decreased from baseline: IL-1β, IL-8, Inflammatory Biomarker Score; and these outcomes increased: IL-6, Leptin, TNF-α. IL-10 and APN decreased in group 2 only. |
| **Duggan** (2016)  USA  RCT | **Mean age:** 57.9 ± 5.0  **Baseline mean BMI:** 30.9 ± 4.0  **Sample size:** 439  **Inclusion criteria:**  **BMI**: Overweight (BMI 25-29.9 kg/m^2^) and higher  **Co-morbidities:** NA | **Lifestyle intervention:**   - Group 1: Reduced-calorie weight loss diet - Group 2: Moderate- to-vigorous intensity aerobic exercise - Group 3: Combined diet and exercise intervention - Group 4: Control | 12 months | Intervention end | **Inflammatory biomarkers**  Oxidized LDL, fluorescent oxidation products and F_2_-isoprostanes | Results were stratified by no change, lost <5% and lost ≥5%. For the lost <5% group there was a significant increase for FOP in group 1 and 3; significant decrease for F_2_-isoprostanes for group 2. No other outcomes were significant (LDL and F_2_-isoprostanes had non-significant decreases for each intervention group) |
| **Falchi** (2014)  Italy  RCT | **Mean age:**  Males: 47.2 ± 12  Females: 49.1 ± 12  **Baseline mean BMI:**  Males: 32.6 ± 2  Females: 35.3 ± 2  **Sample size:** 20  **Inclusion criteria:**  **BMI**: Obesity class 1 (BMI 30-34.9 kg/m^2^) and higher  **Co-morbidities:** NA | **Lifestyle interventions**   - Group 1: Cognitive behavioural therapy + hypocaloric balanced diet - Group 2: Hypocaloric balanced diet only | 6 months | Intervention end | **Cardiovascular**  P wave dispersion | Results were stratified into weight decreased <5% and ≥5%. The <5% weight loss group had no significant changes, but there was a non-significant decrease in males. |
| **Georgoulis** (2022)  Greece  RCT | **Mean age:** 49 ± 10  **Baseline mean BMI:** 35.4 ± 5.9  **Sample size:** 180  **Inclusion criteria:**  **BMI**: Overweight (BMI 25-29.9 kg/m^2^) and higher  **Co-morbidities:** Moderate-to-severe obstructive sleep apnea | **Lifestyle intervention:**   - Group 1: A Mediterranean diet group (diet) - Group 2: A Mediterranean lifestyle group (diet + exercise)   All groups were prescribed with continuous positive airway pressure therapy as the standard care for obstructive sleep apnea management, while the two intervention arms were additionally subjected to a 6-month dietitian-led behavioral intervention. | 6 months | Intervention end | **Quality of life**  AHI, NREM-AHI, REM-AHI, HI, AI ODI and SaO_2_ | Results were stratified by weight-stable/gain group, <5% weight loss group, 5-10% weight loss group and ≥ 10% weight loss group. Within the <5% weight loss group there was a significant decrease in AHI events. Non-significant decrease in NREM-AHI, REM-AHI, AI and ODI events as well as SaO_2_ < 90%. Non-significant increase in HI and minimum SaO_2_. |
| **Gomez-Huelgas** (2019)  Spain  Open-label, non-randomized, intervention study | **Mean age:** 44.5 ± 3.6  **Baseline mean BMI (<5% group):** 35.5 ± 3.6  **Sample size:** 115  **Inclusion criteria:**  **BMI**: Obesity class 1 (BMI 30-34.9 kg/m^2^) and higher  **Co-morbidities:** Metabolic syndrome | **Lifestyle intervention:**   - Hypocaloric Mediterranean diet and a physical exercise program. | 24 months | Intervention end  Additionally, for APN and inflammatory markers 3 & 12 months. | **Anthropometric**  WC  **Cardiovascular**  SBP, DBP, TC, TG, LDL-C, HDL-C  **Metabolic**  Glucose, HbA1c, APN  **Inflammatory biomarkers**  Resistin, hs-CRP, IL-6, TNF-α | Results were stratified by <5% weight loss group, 5-10% weight loss group and ≥ 10% weight loss group. After 24 months, <5% weight loss group noted non-significant decrease in WC, DBP, HbA1c, HDL-C. Non-significant increase in SBP, TC, LDL-C. Significant decrease in glucose, APN, hs-CRP, IL-6 and TNF-α. Significant increase in resistin. No significant change in TG. |
| **Grandi** (2019)  Brazil  Non-randomised intervention study | **Mean age:** 47.4 ± 7.8  **Baseline mean BMI:** 37.9 ± 2.5  **Sample size:** 51  **Inclusion criteria:**  **BMI**: Obesity class 2 (BMI 35-35.9 kg/m^2^) and higher  **Co-morbidities:** Asthma | **Lifestyle intervention:**   - Weight loss program composed of nutritional orientation, psychology support, and an exercise program that included stretching, aerobic, and resistance exercises. | 3 months | Intervention end | **Anthropometric**  WC, fat mass, %body fat, trunk fat mass and visceral fat area  **Metabolic**  APN and leptin  **Inflammatory biomarkers**  CRP, Airway inflammation, interleukins, vascular endothelial growth factor, vitamin D  **Quality of life**  AQLQ domains and asthma control score  **Pulmonary function**  FRC, ERV, RV, IC, TLC, FEV1, FVC,  FEV1/FVC ratio  **Muscle strength** Quadriceps muscle strength and endurance | Results were stratified into weight decreased <5% and ≥5%. In the <5% group, participants showed significant reductions in: WC fat mass, body fat percentage, trunk fat mass and visceral fat area; one domain in AQLQ (environmental stimuli) and asthma control score. There were no changes in airway and systemic inflammation, pulmonary function, or quadriceps muscle function. |
| **Habermann** (2015)  USA  RCT | **Mean age:** 58.2 ± 5.3  **Baseline mean BMI:** 30.6 ± 4.0  **Sample size:** 439  **Inclusion criteria:**  **BMI**: Overweight (BMI 25-29.9 kg/m^2^) and higher  **Co-morbidities:** NA | **Lifestyle intervention:**   - Group 1: Reduced-calorie weight loss diet - Group 2: Moderate- to-vigorous intensity aerobic exercise - Group 3: Combined diet and exercise intervention - Group 4: Control | 12 months | Intervention end | **Metabolic**  DNA repair capacity and radiation sensitivity | Results were stratified into weight decreased <5% and ≥5%. In the <5% group, there was no change in DNA repair capacity or radiation sensitivity compared to baseline or control. |
| **Harrigan** (2016)  USA  RCT | **Mean age (SD):** 59 (7.5)  **Baseline mean BMI (SD):** 33.1 (6.6)  **Sample size:** 100  **Inclusion criteria:**  **BMI**: Overweight (BMI 25-29.9 kg/m^2^) and higher  **Co-morbidities:** NA | **Lifestyle intervention:**   - Group 1: in-person weight loss counseling - Group 2: telephone weight loss counseling - Group 3: usual care | 6 months | Intervention end | **Metabolic**  Insulin, glucose, leptin, and APN  **Inflammatory biomarkers**  IL-6, TNF and CRP | Results were stratified by weight loss <5% and ≥5%.  In the participants who lost <5% weight, there were improvements in insulin, glucose, CRP, leptin and adiponectin (significance not tested). |
| **Höchsmann** (2021)  USA  Cluster RCT | **Mean age:** 49.4 ± 13.1  **Baseline mean BMI:** 37.2 ± 4.7  **Sample size:** 803  **Inclusion criteria:**  **BMI**: Obesity class 1 (BMI 30-34.9 kg/m^2^) and higher  **Co-morbidities:** NA | **Lifestyle intervention:**   - Group 1: Intensive lifestyle interventions (ILI) group received a comprehensive, high-intensity lifestyle intervention program. Participants developed action plans focusing on changes in eating, diet, and PA behavior. - Group 2: Usual care (control) | 24 months | 12 months & intervention end | **Cardiovascular**  TC, TG, LDL-C, HDL-C, non-HDL-C, total HDL-C ratio, SBP, DBP, and mean arterial pressure  **Metabolic**  Fasting glucose, metabolic syndrome severity Z-score | Results were stratified by <5% weight loss group, 5-10% weight loss group and ≥ 10% weight loss group. Within the <5% weight loss group HDL-C at 12 months was the only significant difference (increase) amongst the Cardiovascular and metabolic markers. |
| **Imayama** (2012)  USA  RCT | **Mean age:**  Group 1: 58.1 ± 5.9  Group 2: 58.1 ± 5.0  Group 3: 58.0 ± 4.4  Group 4: 57.4 ± 4.4  **Baseline mean BMI:**  Group 1: 31.1 ± 3.9  Group 2: 30.7 ± 3.7  Group 3: 31.0 ± 4.3  Group 4: 30.7 ± 3.9  **Sample size:** 439  **Inclusion criteria:**  **BMI**: Overweight (BMI 25-29.9 kg/m^2^) and higher  **Co-morbidities:** NA | **Lifestyle intervention:**   - Group 1: Caloric restriction diet with a goal of 10% weight reduction. - Group 2: Moderate-to-vigorous intensity aerobic exercise for 45 minutes/day, 5 days/week. - Group 3: Combined exercise and diet. - Group 4: Control | 12 months | Intervention end | **Inflammatory biomarkers**  H-CRP, SAA, IL6, leukocytes, and neutrophils | Results were stratified into weight decreased <5% and ≥5%. In the <5% weight loss group there was a significant decrease in leukocytes, but not in any other inflammatory biomarkers. The analyses were adjusted for baseline BMI, ethnicity, age. |
| **Johnson** (2011)  USA  RCT | **Mean age (SD):** 49.5 (11.0)  **Baseline mean BMI (SD):** 46.1 (8.0)  **Sample size:**  208  **Inclusion criteria:**  **BMI**: NA  **Co-morbidities:** NA | **Pharmacological and lifestyle intervention:**   - Group 1: Intensive medical weight loss intervention – phase 1 = liquid hypocaloric diet, phase 2 = sibutramine or diethylpropion hydrochloride and orlistat, a hypocaloric diet and group counselling sessions, phase 3 = continuation of phase 2 recommended - Group 2: Usual care | Phase 1 = 12 weeks; Phase 2 = 4 months; Phase 3 = 16 months | 12 months | **Cardiovascular**  SBP, DBP, HDL, LDL, TG  **Metabolic**  FPG  **Renal and hepatic**  Uric acid | Results were stratified by weight change: gain >2%, stable ±2%, minimal loss 2-4.9%, modest loss 5-9.9%, substantial loss 10-19.9%, big loss ≥20%. For the participants who lost 2-4.9%, there was an improvement in SBP, DBP, TG, and Uric acid but a worsening in FPG, HDL (significance not tested). |
| **Jouneau** (2020)  24 countries  RCT | **Mean age:** 66.8 ± 8.0  **Baseline mean BMI:** 27.9 ± 4.6  **Sample size:** 638  **Inclusion criteria:**  **BMI**: NA  **Co-morbidities:** Idiopathic pulmonary fibrosis | **Pharmacological intervention:**   - Group 1: Nintedanib (150 mg) twice daily. - Group 2: Control | 52 weeks | Intervention end | **Pulmonary function**  FVC, and St. George’s Respiratory Questionnaire | Results were stratified into weight loss of ≤5% and >5%. In the participants who lost ≤5% weight, those in group 1 had a significantly lower decline in FVC, compared to group 2 (control); but no significant difference in the St. George’s Respiratory Questionnaire score. |
| **Kaholokula** (2013)  USA  RCT | **Mean age:** 50.4 ± 14.7  **Baseline mean BMI:** NR  **Sample size:** 100  **Inclusion criteria:**  **BMI**: Overweight (BMI 25-29.9 kg/m^2^) and higher  **Co-morbidities:** NA | **Lifestyle intervention:**  All participants completed a 3-month weight loss program (WLP) to initiate weight loss.   - Group 1: 6-month family/community focused WLP called the PILI Lifestyle Program - Group 2: a standard behavior WLP (control) | 9 months | 3 month & intervention end | **Cardiovascular**  SBP and DBP  **Psychosocial and behavioral**  Exercise fatigue level, 6-min walk test, exercise frequency and fat in diet | Results were stratified into weight loss of <3% and ≥3% in the first 3-months of the study. In the <3% group, the changes from baseline were not tested. The observed results were improved in the 6-min walk test results, SBP and DBP. |
| **Kiddy** (1992)  UK  Intervention | **Mean age:** NR  **Baseline mean BMI (SD):** 34.1 (4.9)  **Sample size:** 24  **Inclusion criteria:**  **BMI**: Obesity class 1 (BMI 30-34.9 kg/m^2^) and higher  **Co-morbidities:** Polycystic ovary syndrome | **Lifestyle intervention:**  All participants were scheduled for a low-calorie, low-fat diet.   - Those with a BMI greater than 30 kg/m^2^ were initially given the option of a very low-calorie diet (330 kcal per day) for 4 weeks, followed by a 1000 kcal per day low fat diet for a further 6 months. - Those with a BMI between 25 and 30 kg/m^2^ and those who opted against the very low-calorie diet started on the 1000 calorie low fat diet. | 7 months | Intervention end | **Metabolic**  Testosterone, free testosterone, fasting insulin, sum of insulin concentrations, sex hormone-binding globulin, and IGF-1  **Ovulatory function** | Results were stratified into weight loss of <5% and >5%. Within the <5% weight loss group, there was a non-significant decrease in free testosterone, sex hormone-binding globulin, and fasting insulin, while non-significant increase in sum of insulin concentrations, and IGF-1. Additionally, only one of the eight with menstrual disturbances noted an improvement in reproductive function and none had a significant reduction in hirsutism. |
| **Kolehmainen** (2008)  Finland  RCT | **Mean age (SD):**  Group 1: 59 (7)  Group 2: 61 (7)  **Baseline mean BMI (SD):** Group 1: 32.9 (3.2)  Group 2: 32.4 (2.5)  **Sample size:**  46  **Inclusion criteria:**  **BMI**: Overweight (BMI 25-29.9 kg/m^2^) and higher  **Co-morbidities:** Metabolic syndrome | **Lifestyle intervention**   - Group 1: Intensive weight reduction program with individual nutrition counselling - Group 2: Control | 12 weeks | 33 weeks | **Anthropometric**  WC, body fat % and mass (kg), lean body mass (kg)  **Metabolic**  FPG, 2h-PG, FSIGT, insulin sensitivity, glucose effectiveness, acute insulin response, FS leptin, adipocyte cell size | Results were stratified by weight loss <5% and ≥5%. In the participants who lost <5% weight, there were significant decreases in WC, body fat mass, FPG, FS leptin (significant). There were non-significant decreases in lean body mass, 2h-PG and glucose effectiveness; non-significant increases in FSIGT, insulin sensitivity and acute insulin response. |
| **Kolotkin** (2009)  USA  RCT | **Mean age:** 49.5 ± 11.1  **Baseline mean BMI:** 35.4 ± 3.8  **Sample size:** 926  **Inclusion criteria:**  **BMI**: Obesity class 1 (BMI 30-34.9 kg/m^2^) and higher  **Co-morbidities:** Obesity-related | **Lifestyle and pharmacological intervention:**   - Group 1: Experimental drug + two week diet and exercise run in and hypocaloric diet + diet and exercise training - Group 2: Same as above, except placebo drug not experimental | 12 months | Intervention end | **Quality of life**  IWQOL-Lite scores, EQ-5D scores and SF-36 scores | Results were stratified by ≥ 5% gain, 0 to 4.9 gain, 0 to 4.9 loss, 5 to 9.9% loss and ≥10% loss. In the 0-4.9% loss group, there was an improvement in IWQOL total score (small but significant effect size). Worsening SF-36 components (mental component, bodily pain, general health, vitality, social functioning, role emotional and mental health). No significant changes in EQ-5D. |
| **Konerman** (2019)  USA  Non-randomised intervention study | **Median age:** 54  **Baseline median BMI [IQR]:** 36.9 [32.6, 42.9]  **Sample size:** 403  **Inclusion criteria:**  **BMI**: NA  **Co-morbidities:** Metabolic syndrome and non-alcoholic fatty liver disease | **Lifestyle intervention:**   - Metabolic Fitness (MetFit) Programme | 12 weeks or 24 weeks | 12 weeks & 24 weeks | **Cardiovascular**  TG, HDL-C, LDL-C  **Metabolic**  HbA1c, fasting glucose, fasting insulin, HOMA-IR, and metabolic syndrome components  **Renal and hepatic**  ALT  **Psychosocial and behavioral**  PHQ-9 depression score | Results were stratified into weight loss of <5% and ≥5%. The <5% weight loss group had improvements in all features of metabolic syndrome (significance not tested). There were non-significant improvements in A1c, fasting glucose and insulin, TG, LDL-C and ALT. |
| **Kosiborod** (2022)  USA  Placebo‐controlled trials | **Mean age:**  Group 1: 46 ± 13  Group 2: 47 ± 12  **Baseline mean BMI:**  Group 1: 37.8 ± 6.7  Group 2: 38.0 ± 6.5  **Sample size:**  1,961  **Inclusion criteria:**  **BMI**: Overweight (BMI 25-29.9 kg/m^2^) and higher  **Co-morbidities:** Obesity-related | **Lifestyle and pharmacological intervention:**   - Group 1: Once weekly s.c. semaglutide (2.4 mg) with lifestyle intervention. - Group 2: Placebo | 68 weeks | Week 20 & intervention end | **Anthropometric**  WC  **Cardiovascular**  SBP, DBP, non-HDL, LDL, and TG  **Metabolic**  FPG, fasting serum insulin, and HOMA‐IR | Results were stratified by weight loss <5%, 5% to <10%, 10% to <15%, and ≥15%. In the <5% group, there were decreases in SBP, WC, TG, FPG (group 1 only) and increases in HOMA-IR, non-HDL, LDL, fasting serum insulin and DBP (Group 1 only). |
| **Lang** (2011)  China  Clinical trial | **Mean age:** 40.3 ± 10.8  **Baseline mean BMI:** 30.0 ± 3.4  **Sample size:** 14  **Inclusion criteria:**  **BMI**: Overweight (BMI 25-29.9 kg/m^2^) and higher  **Co-morbidities:** NA | **Lifestyle intervention:**  Weight-control program that combined dietary guidance and aerobic exercise training. | 8 weeks | Intervention end | **Anthropometric**  WC, hip circumference, body fat mass, visceral fat mass, body fat composition, subcutaneous fat mass  **Cardiovascular**  DBP, TC, LDL-C and HDL-C  **Metabolic**  APN  **Inflammatory biomarkers**  TNF-α | Results were stratified by weight loss <3%, and weight loss ≥3%. Within the <3% weight loss group there was a non-significant decrease in DBP, WC, body fat mass, subcutaneous fat mass, TC, LDL-C, HDL-C and TNF-α. Non-significant increase was noted in body fat composition and APN, furthermore no change was noted in visceral fat mass. There was statistically significant decrease in hip circumference. |
| **Magkos** (2017)  USA  RCT | **Mean age (<5% weight loss):**  Group 1: 52.2 ± 8.6  Group 2: 52.1 ± 9.3  **Baseline mean BMI (<5% weight loss):**  Group 1: 36.5 ± 4.4  Group 2: 35.8 ± 4.6  **Sample size:** 434  **Inclusion criteria:**  **BMI**: Overweight (BMI 25-29.9 kg/m^2^) and higher  **Co-morbidities:** Diabetes | **Lifestyle and pharmacological interventions:**   - Group 1: Received Lorcaserin (10 mg) twice daily. - Group 2: Received placebo twice daily.   Additionally, all participants were instructed to exercise for 30 min/d and reduce their daily caloric intake by 600 kcal below estimated daily energy requirements. | 12 months | 12 weeks & intervention end | **Metabolic**  HbA1c, fasting plasma glucose, fasting serum insulin, HOMA-IR | Results were stratified by weight loss <5%, and weight loss ≥5%. Participants receiving Lorcaserin within the <5% weight loss group had a greater reduction in HbA1c and fasting plasma glucose than those receiving the placebo. At 12 months, participants receiving Lorcaserin within the <5% weight loss group had a greater decrease in fasting insulin than those receiving placebo. Lastly, participants receiving Lorcaserin also had greater reduction in HOMA-IR at all time points, compared to participants receiving placebo. Significance not tested. |
| **Maruthur** (2013)  USA  RCT | **Median age [IQR]:**  Group 1: 50 [42,59]  Group 2: 51 [44,57]  Group 3: 50 [44,57]  **Baseline mean BMI:** NR  **Sample size:** 3,041  **Inclusion criteria:**  **BMI**: NA  **Co-morbidities:** Diabetes | **Lifestyle and pharmacological interventions:**   - Group 1: Intensive lifestyle modification - Group 2: Metformin was started at 850 mg by mouth once daily and increased to twice daily - Group 3: Placebo | 12 months | 6 months & intervention end | **Metabolic**  Diabetes risk | Results were stratified by weight loss <0%, weight loss 0 to <3%, weight loss 3 to <5%, weight loss 5 to <7%, weight loss 7 to <10% and weight loss ≥10%. Within the 0 to <3% weight loss group, there was a non-significant decrease in the risk of diabetes for group 1, 2 and 3. Similarly all 3 intervention groups noted a non-significant decrease within the 3 to <5% weight loss group. |
| **Mason** (2015)  USA  RCT | **Mean age:** 57.9 ± 5.0  **Baseline mean BMI:** 30.9 ± 4.0  **Sample size:** 439  **Inclusion criteria:**  **BMI**: Overweight (BMI 25-29.9 kg/m^2^) and higher  **Co-morbidities:** NA | **Lifestyle intervention:**   - Group 1: Dietary weight loss. - Group 2: Moderate-to-vigorous intensity aerobic exercise. - Group 3: Combined exercise and diet. - Group 4: Control (no intervention) | 12 months | Intervention end | **Metabolic**  Fasting total serum ghrelin | Results were stratified by ≤0% loss, <5% loss, 5-10% loss and ≥10% loss. For the <5% loss group there was a non-significant increase for total ghrelin in intervention group 2 and 3; significant decrease for total ghrelin in intervention group 1. |
| **Messier** (2009)  Canada  RCT | **Mean age:** Group 1: 58.0 ± 4.7  Group 2: 57.2 ± 5.0  **Baseline mean BMI:**  Group 1: 32.2 ± 4.6  Group 2: 32.6 ± 4.9  **Sample size:** 137  **Inclusion criteria:**  **BMI**: Overweight (BMI 25-29.9 kg/m^2^) and higher  **Co-morbidities:** NA | **Lifestyle intervention:**   - Group 1: Caloric restriction. - Group 2: Caloric restriction + resistance training. | 6 months | Intervention end | **Psychosocial and behavioral**  Total body esteem, appearance, attribution, self-esteem, stress, dietary restraint, disinhibition, hunger  **Quality of life**  Physical functioning, pain, social functioning, role functioning, mental health, health perceptions, self-efficacy, perceived risk for heart disease and diabetes | Results were stratified by weight change + 5.3 to −2.3%, −2.4 to −4.8%, −4.9 to −8.3%, −8.4 to −11.0% and −11.1 to −18.1%. Within the – 2.4 to – 4.8% weight change group, there was a significant increase in total body esteem, appearance, attribution, and dietary restraint. Significant decrease was noted in self-esteem and hunger, whereas non-significant decrease was noted in stress. Non-significant increase in disinhibition. Overall, the total quality of life observed a slight non-significant decrease. |
| **Miazgowski** (2021)  Poland  Intervention study | **Mean age:** 36.73 ± 7.2  **Baseline mean BMI:** 35.8  **Sample size:** 111  **Inclusion criteria:**  **BMI**: NA  **Co-morbidities:** Metabolic syndrome or type 2 diabetes | **Lifestyle intervention:**   - An approximately 2-h session with a doctor and a trained dietician, participants were encouraged to implement healthier behaviors and received written advice targeting lifestyle modification according to current guidelines. (diet + exercise) | 4 months | Intervention end | **Anthropometric**  Fat, android fat, gynoid fat, visceral fat, and lean mass  **Cardiovascular**  LDL-C, HDL-C, TG  **Metabolic**  Glucose, Insulin, HOMA-IR, Irisin  **Renal and hepatic**  Uric acid, ALT | Results were stratified by no weight loss, <5% weight loss, and >5% weight loss. Within the <5% weight loss group, there was a statistically significant decrease in glucose, insulin, HOMA-IR, TG, ALT, uric acid and all the anthropometric measures. Non-significant decrease was noted in irisin and LDL-C. Lastly, HDL-C noted an increase however significance was not discussed. |
| **Muls** (2001)  Belgium  RCT | **Mean age (SD):**  Group 1: 49.6 (10)  Group 2: 47.5 (11)  **Baseline mean BMI (SD):**  Group 1: 32.9 (3.5)  Group 2: 33.0 (3.7)  **Sample size:** 294  **Inclusion criteria:**  **BMI**: Overweight (BMI 25-29.9 kg/m^2^) and higher  **Co-morbidities:** Metabolic syndrome | **Pharmacological and lifestyle interventions**   - Group 1: Orlistat 120 mg three times daily and hypocaloric diet - Group 2: Placebo and hypocaloric diet | 24 weeks | Intervention end | **Cardiovascular**  LDL | Results were stratified by weight loss <0%, 0-2.5%, 2.5-5%, 5-7.5%, 7.5-10% and >10%. For the participants in group 1 who lost 0-2.5% and those in group 1 and 2 who lost 2.5-5% weight, there was a decrease in LDL; and there was an increase in LDL for those who lost 0-2.5% in group 2 (significance not tested). |
| **Muramoto** (2014)  Japan  Controlled clinical trial | **Mean age:** 48.3 ± 5.9 years  **Baseline mean BMI:** 27.7 ± 2.5  **Sample size:** 3,480  **Inclusion criteria:**  **BMI**: Overweight (BMI 25-29.9 kg/m^2^) and higher  **Co-morbidities:** Metabolic syndrome | **Lifestyle intervention:**   - Participants received detailed information of their health examination data and attended a lecture about obesity-associated health problems and the benefits of weight reduction. Participants set their own behavioural goals. | 12 months | Intervention end | **Anthropometric** Abdominal circumference  **Cardiovascular**  SBP, DBP, TG, LDL-C, HDL-C  **Metabolic**  Fasting plasma glucose, HbA1c  **Renal and hepatic**  AST, ALT, γGTP and uric acid | Results were stratified by weight loss ≥3% and <3%. Within the <3% weight reduction group, improvement was observed for all the outcomes that were measured. Significance between baseline and follow-up values was not tested. However, all the parameters significantly improved in the ≥3% weight reduction group compared to <3% group. |
| **Nadinskaia** (2021)  Russia, Kazakhstan and Uzbekistan  Noncomparative clinical trial | **Mean age:** 45.2 ± 10.1  **Baseline median BMI [IQR]:** 31.2 [29.4, 34.0]  **Sample size:** 183  **Inclusion criteria:**  **BMI**: NA  **Co-morbidities:** Non-alcoholic fatty liver disease | **Lifestyle and pharmacological intervention:**   - All participants received Ursodeoxycholic acid (15 mg/kg body weight) daily for 6 months. In addition, standard lifestyle recommendations were given, including, strength or aerobic exercise for at least 150 min per week, Mediterranean diet, and consumption of no more than 1500 kcal/day. | 6 months | 3 months & intervention end | **Cardiovascular**  TC, TG, LDL-C  **Renal and hepatic**  AST, ALT, GGT, fatty liver index | Results were stratified by weight loss >5%, and ≤5%. Within the ≤5% weight loss group at 6 months, there is a non-significant decrease in ALT, AST, GGT, TC, TG, and LDL-C. There was a statistically significant decrease in fatty live index. |
| **Nagahara** (2021)  Japan  Intervention | **Age range:** 40-64  **Baseline mean BMI (SD):** NR  **Sample size:** 5,031  **Inclusion criteria:**  **BMI**: NA  **Co-morbidities:** NA | **Lifestyle intervention**   - Group 1: Active support for specific health guidance following health checkup – behavioural goal setting with health professionals with monthly support (call, email, letter) | 6 months | 3,6 months and following years check up | **Anthropometric**  WC  **Cardiovascular**  SBP, DBP, HDL-C, TG  **Metabolic (following years check-up)**  Fasting glucose, Met-S incidence and escape rate | Results were stratified into no weight loss, 0–<3% loss, ≥3% loss. For the participants who lost <3%, WC, SBP and DBP all significantly decreased in the at 3 and 6 months, compared to baseline. For the same participants, WC, SBP, DBP, HDL-C and TG all significantly decreased from the initial vs the following year check-up. The Met-S incidence of the <3% weight loss group was 12.3% compared to 18.9% in the non-weight loss group; and the Met-S escape rate was 53.7% and significant. |
| **Patrick** (2003)  Study 2 -  USA  Secondary analysis of clinical trial | **Mean age:** 44.5 ± 10.7  **Baseline mean BMI:** 37.3 ± 5.2  **Sample size:** 1,282  **Inclusion criteria:**  **BMI**: NA  **Co-morbidities:** NA | **Pharmacological intervention:**  A clinical trial was conducted to evaluate a product for weight loss among obese persons without a diagnosis of diabetes | 50-83 weeks | 50-83 weeks | **Quality of life**  OWLQOL score and WSRM bother scores | Results were stratified into weight increase, 0 to 4.99% decrease, 5 to 9.99% decrease and ≥10.0% decrease. In the 0 to 4.99% group the OWLQOL score and WSRM bother scores both improved. Significance was not tested. |
| **Perreault** (2008)  USA  RCT | **Mean age:**  ILS group:  Males: 54.0 ± 17.4  Females: 47.8 ± 14.5  Placebo group:  Males: 53.1 ± 15.2  Females: 48.3 ± 12.9  **Baseline mean BMI:**  ILS group:  Males: 30.9 ± 6.2  Females: 33.6 ± 8.6  Placebo group:  Males: 30.9 ± 7.1  Females: 34.1 ± 9.6  **Sample size:** 2,161  **Inclusion criteria:**  **BMI**: Overweight (BMI 25-29.9 kg/m^2^) and higher  **Co-morbidities:** NA | **Lifestyle and pharmacological intervention:**   - Group 1: Placebo twice daily and standard lifestyle recommendations. - Group 2: Metformin (850 mg twice daily), and standard lifestyle recommendations. - Group 3: Intensive lifestyle modification (ILS) a healthy low-calorie, low-fat diet and to engage in physical activity of moderate intensity. | 12 months | Intervention end | **Anthropometric**  WC  **Cardiovascular**  BP, TG, HDL-C  **Metabolic**  Fasting glucose, 2-h glucose, HbA1C, insulin, carbohydrate‐to‐insulin ratio, and HOMA-IR | Results were stratified by gender as well as by <3% weight loss, 3-7% weight loss and >7% weight loss. Within the <3% weight loss group there was a non-significant decrease in WC, fasting glucose, 2-h glucose, insulin, and HOMA-IR for both men and women. There was a non-significant increase in BP for both men and women. There was a non-significant increase in TG and carbohydrate‐to‐insulin ratio among women and a non-significant decrease among men. There was no change noted for HDL among men, however there was a non-significant decrease among women. Lastly, there was a non-significant decrease in A1C among men and no change among women. |
| **Poppitt** (2002)  UK  Clinical trial | **Mean age:**  Group 1: 45.9 ± 5.0  Group 2: 44.2 ± 5.5  Group 3: 48.6 ± 4.4  **Baseline mean BMI:**  Group 1: 30.9 ± 3.0  Group 2: 32.3 ± 3.6 Group 3: 33.1 ± 3.3  **Sample size:** 46  **Inclusion criteria:**  **BMI**: Overweight (BMI 25-29.9 kg/m^2^) and higher  **Co-morbidities:** NA | **Lifestyle intervention:**   - Group 1: the low-fat, high-simple-carbohydrate diet. - Group 2: the low-fat, high-complex-carbohydrate diet. - Group 3: Control diet. | 6 months | Intervention end | **Anthropometric**  WC  **Cardiovascular**  TC, TG, HDL-C, LDL-C, SBP, DBP | Results were stratified by <3% weight loss, 3–7% weight loss and >7% weight loss. Within the <3% weight loss group, there was a non-significant decrease in WC, TC, HDL-C, LDL-C and SBP. TG and DBP noted a non-significant increase. |
| **Rintamaki** (2021)  Finland  Cohort study | **Mean age (2.5−4.9% weight loss):** 55.8 ± 9.8  **Baseline mean BMI (2.5−4.9% weight loss):** 31.5 ± 4.9  **Sample size:** 8,353  **Inclusion criteria:**  **BMI**: NA  **Co-morbidities:** Metabolic syndrome, cardiovascular disease or gestational diabetes | **Lifestyle intervention:**  Either individual or group-based lifestyle counselling, discussing weight reduction, a healthy diet and physical activity based on the persons individual needs. There was no uniform protocol for the frequency and intensity of the intervention. | 7.4 years | Intervention end | **Cardiovascular**  CVD events  **Metabolic**  Diabetes incidence    **Total mortality** | Results were stratified by weight loss ≥5%, weight loss 2.5−4.9%, stable weight and gained weight. Within the 2.5-4.9% weight loss group, the risk of diabetes incidence was significantly lower (HR = 0.63, P<0.001) compared to baseline, and there were no significant changes for CVD events or total mortality. These analyses were adjusted for age, sex, BMI, fasting glucose, 2-h glucose, drug treatment for hypertension and drug treatment for lipids at baseline. |
| **Rock** (2013)  USA  Intervention study | **Mean age:**  55 ± 10 years  **Baseline mean BMI:** NR  **Sample size:** 258  **Inclusion criteria:**  **BMI**: Overweight (BMI 25-29.9 kg/m^2^) and higher  **Co-morbidities:** NA | **Lifestyle intervention:**   - Group 1: Immediate intervention – promoting regular physical activity, a modest reduction in energy intake, and healthy eating attitudes and behaviors. - Group 2: Wait-list control group. | 18 months | 6 months, intervention end | **Metabolic**  Insulin, leptin, and sex hormone binding globulin | Results were stratified by weight loss ≥5%, and weight loss <5%. Within the <5% weight loss group at both 6 and 18 months, there was an increase in insulin, and a decrease in leptin and sex hormone-binding globulin. Significance was not tested. |
| **Rusu** (2013)  Romania  RCT | **Mean age:**  Group 1:  Males: 52.5 ± 7.1  Females: 55.4 ± 9.7  Group 2:  Males: 52 ± 8.4  Females: 54.9 ± 10.1  **Baseline mean BMI:**  Group 1:  Males: 30.6 ± 4.4  Females: 29.6 ± 2.3  Group 2:  Males: 30.7 ± 4.2  Females: 28.5 ± 2.4  **Sample size:** 120  **Inclusion criteria:**  **BMI**: Overweight (BMI 25-29.9 kg/m^2^) and higher  **Co-morbidities:** Chronic hepatitis C | **Lifestyle intervention:**   - Group 1: Normoglucidic low-calorie diet (NGLCD) - Group 2: Low-fat diet (LFD)   All participants were required to limit alcohol intake and conduct moderate intensity physical activities. | 18 months | 6 months & intervention end | **Renal and hepatic**  AST, ALT, Albumin, Bilirubin, GGT, AST/ALT, FI and APRI | Results were stratified by weight gain, 1-5% weight loss, 5-10% weight loss and weight loss >10%. In participants with less than 5% loss of weight within the NGLCD group noted improvements in ALT levels, GGT levels, AST/ALT ratio and FI. Similarly, participants with less than 5% loss of weight within the LFD group noted improvements in AST, ALT, total bilirubin, INR, FI, APRI. Significance was not tested. |
| **Scott** (2012)  Australia  RCT | **Mean age:**  40.3 ± 13.4  **Baseline mean BMI:** 33.7 ± 3.5  **Sample size:** 46  **Inclusion criteria:**  **BMI**: Overweight (BMI 25-29.9 kg/m^2^) and higher  **Co-morbidities:** Asthma | **Lifestyle intervention:**   - Group 1: Dietary intervention - Group 2: Physical activity intervention - Group: Diet + physical activity intervention | 10 weeks | Intervention end | **Pulmonary function**  FEV1, FVC, FEV1/FVC ratio, TLC, FRC, ERV, RV, ACQ, and AQLQ | Results were stratified by weight loss <5%, 5-10%, and >10%. Within the <5% weight loss group, significant decrease was noted for ACQ, and significant increase was noted for AQLQ. The remaining characteristics had non-significant differences. |
| **Sharma** (2009)  Canada  RCT | **Mean age [IQR]:**  Group 1: 62.0 [56.0, 75.0]  Group 2: 63.0 [56.0, 74.0]  Group 3: 63.0 [56.0, 75.0]  **Baseline mean BMI [IQR]:** Group 1: 33.0 [27.8, 41.9]  Group 2: 33.7 [28.1, 43.0]  Group 3: 34.3 [28.3, 43.6]  **Sample size:** 10,742  **Inclusion criteria:**  **BMI**: NA  **Co-morbidities:** Cardiovascular disease or type 2 diabetes | **Lifestyle and pharmacological intervention:**   - Sibutramine (10 mg) with advice on an individualized caloric-deficit diet and a moderate exercise programme - Participants were stratified by blood pressure at baseline: group 1 = normal; group 2 = high-normal; group 3 = hypertensive. | 6 weeks | Intervention end | **Cardiovascular**  SBP, DBP | Results were stratified into gain or no loss, weight loss >0 – 2.5%, >2.5% - 5%, and >5%. In the >2.5 to 5% weight loss group there was a significant decrease in both SBP and DBP for all patients. For the >0 – 2.5% weight loss groups there was a significant decrease in SBP and DBP for all participants, except for participants classified as having high-normal BP at baseline and taking 1 antihypertensive medication. |
| **Sheng** (2022)  USA  RCT | **Median age [IQR] (< 5% weight loss):** 54 [30, 73]  **Baseline median BMI [IQR] (< 5% weight loss):** 31.6 [26.2, 45.3]  **Sample size:** 96  **Inclusion criteria:**  **BMI**: Overweight (BMI 25-29.9 kg/m^2^) and higher  **Co-morbidities:** NA | **Lifestyle intervention:**   - Group 1: POWER-remote intervention (telephone coaching; diet/activity tracking) - Group 2: Self-directed weight-loss (booklet) | 12 months | 6 months & intervention end | **Psychosocial and behavioral**  Physical function, sexual function, endocrine symptoms, pain interference, fatigue, depression, anxiety, and sleep disturbance | Results were stratified by weight loss at 6 months and 12 months, as well as weight loss <5% and ≥5%. Within the <5% weight loss at 6 months group, there was a non-significant increase in physical function, endocrine symptoms, pain interference, depression. Non-significant decrease in fatigue, anxiety, sleep disturbance. Significant decrease in sexual function. Within the <5% weight loss at 12 months group, no change in physical function; non-significant increase in sexual function, endocrine symptoms, pain interference. Non-significant decrease in fatigue, depression, anxiety, sleep disturbance. |
| **Shirai** (2013)  Japan  Multicenter randomized trial | **Mean age:** 51.1 ± 11.4  **Baseline mean BMI:** 30.4 ± 5.3  **Sample size:** 240  **Inclusion criteria:**  **BMI**: NA  **Co-morbidities:** Type 2 diabetes | **Lifestyle intervention:**   - Group 1: Dietary regimen using conventional diet (CD) - classical Japanese low-caloric meals 3 times a day - Group 2: Dietary regimen using formula diet (FD) - one pack of MicroDiet® (240 kcal/meal) in the morning and two conventional Japanese low-caloric meals at noon and in the evening. | 24 weeks | 8 weeks, 16 weeks & intervention end | **Cardiovascular**  SBP, DPB, TG, HDL-C, LDL-C, non-HDL-C  **Metabolic**  Fasting blood glucose, HbA1C, insulin, HOMA-IR, APN and leptin | Results were assessed per 1% body weight reduction (mean % body weight reduction at week 24 was ~5%, observed from a figure). Significant decreases in SBP, TG, fasting blood glucose and HbA1C, were observed in both interventions groups. Significant decreases in DBP were observed only in FD group between week 4 to 20. LDL-C and non-HDL-C decreased in both groups (significance not reported). HDL-C decreased initially and remained significantly lower than baseline until week 12 in FD and 20 weeks in CD, however, by week 24 HDL-C reached significantly higher level in FD. Insulin decreased significantly in FD only at week 24 and did not decrease in CD. HOMA-IR was significantly lower than baseline in FD but did not change in CD. |
| **Smith** (2011)  USA + Sweden  RCT | **Mean age (SD):** 43.4 (10.4)  **Baseline mean BMI (SD):** 31.0 (2.2)  **Sample size:** 123  **Inclusion criteria:**  **BMI**: Overweight (BMI 25-29.9 kg/m^2^) and higher  **Co-morbidities:** NA | **Pharmacological and lifestyle interventions**   - Group 1: Orlistat 60mg and lifestyle intervention (nutrition counselling, hypocaloric diet and encouraged to exercise) - Group 2: Placebo and lifestyle intervention | 24 weeks | 24 weeks | **Anthropometric**  Visceral adipose tissue | Results were stratified by weight loss <5% and ≥5%. For the participants in group 1 who lost <5% weight, there was a significant decrease in VAT, but no significant change in group 2. |
| **Spurny** (2020)  Germany  RCT | **Mean age:**  ≤2% weight loss: 50.9 ± 6.4  >2% to ≤4.5% weight loss: 50.8 ± 8.4  **Baseline mean BMI:**  ≤2% weight loss: 32.2 ± 4.1  >2% to ≤4.5% weight loss: 30.8 ± 3.6  **Sample size:** 137  **Inclusion criteria:**  **BMI**: Overweight (BMI 25-29.9 kg/m^2^) and higher  **Co-morbidities:** NA | **Lifestyle interventions:**   - Group 1: Intermittent calorie restriction - Group 2: Continuous calorie restriction | 50 weeks | 12 weeks & intervention end | **Metabolic**  Bone marrow fat content | Results were stratified by ≤2% weight loss, >2% to ≤4.5% weight loss, >4.5% to ≤7.5% weight loss, and >7.5% weight loss. Within the ≤2% weight loss group at 12 and 50 weeks there was a significant increase in bone marrow fat content. Whereas, within the >2% to ≤4.5% weight loss group at 12 and 50 weeks there was a significant decrease in bone marrow fat content. |
| **St. George** (2009)  Australia  RCT | **Mean age:**  48.13 ± 11.16  **Baseline mean BMI:** 31.7 ± 5.3  **Sample size:** 185  **Inclusion criteria:**  **BMI**: NA  **Co-morbidities:** Non-alcoholic fatty liver disease or chronic hepatitis C | **Lifestyle interventions:**   - Group 1: Low-intensity (3 sessions/4 weeks) lifestyle counselling and education sessions for 3 months. - Group 2: Moderate-intensity (6 sessions/10 weeks) lifestyle counselling and education sessions for 3 months. - Group 3: Moderate-intensity (6 sessions/10 weeks) lifestyle counselling and education sessions for 3 months. After the 3 month assessment, it was intended that Group 3 receive a telephone-based maintenance program through to 12 months. - Group 4: Control | 3 months | Intervention end | **Renal and hepatic**  ALT, GGT, AST | Results were stratified into weight gain ≥ 0.3%, weight maintenance −1.9% to +0.2%, moderate weight loss -2.0 to -3.9%, and high weight loss ≥4.0%. In the moderate weight loss group, there was a significant reduction in all the liver enzymes from baseline. |
| **Strelitz** (2019)  UK  Cohort analysis -following a cluster-randomised trial | **Mean age** **(SD):** 61.1 (7.1)  **Baseline mean BMI (SD):** 33.4 (5.6)  **Sample size:** 725  **Inclusion criteria:**  **BMI**: NA  **Co-morbidities:** Type 2 diabetes | **Lifestyle interventions**   - Group 1: Intensive treatment - higher frequency consultations, educational materials (including on medications and lifestyle advice) and GP-based academic sessions on risk factors - Group 2: Routine care | 3 years | 1 year and 5 years | **Cardiovascular**  SBP, DBP, TG, TC, HDL-C, LDL-C, CVD incidence    **Metabolic**  HbA1C  **Total mortality** | Results were stratified into >2% gain, maintained weight (≤2% gain or <2% loss), ≥2% to <5% loss, ≥5% to <10% loss, and ≥10% loss. In the participants who lost ≥2 - <5% weight, at 1 and 5 years there were improvements with decreases in SBP, DBP, TC, LDL, TG, HbA1c (percent change, not median); and increases in HDL. Compared to the maintained weight group, the hazard ratio of 10 year CVD incidence was lower but not significant; and greater for all-cause mortality but not significant. |
| **Swift** (2016)  USA  RCT | **Mean age:** 57.3 ± 6.5  **Baseline mean BMI:** 31.9 ± 5.4  **Sample size:** 464  **Inclusion criteria:**  **BMI**: Overweight (BMI 25-29.9 kg/m^2^) and higher  **Co-morbidities:** NA | **Lifestyle intervention:**   - Group 1: Aerobic training at 4 kcal per kilogram per week - Group 2: Aerobic training at 8 kcal per kilogram per week - Group 3: Aerobic training at 12 kcal per kilogram per week. - Group 4: Control | 6 months | Intervention end | **Anthropometric**  WC  **Cardiovascular**  LDL-C, HDL-C, TG, TC, SBP, DBP  **Metabolic**  Glucose, insulin | Results were stratified by ≥5.0% weight loss (CWL), ≥3.0% to ≤5.0% weight loss (MWL) and <3.0% weight loss (No CWL or MWL). Within the MWL group, non-significant decrease was observed in WC, TC, glucose, and insulin. Non-significant increase was observed in HDL-C, LDL-C, TG, SBP and DBP. Within the no CWL or MWL group, non-significant decrease was observed in WC, HDL-C, LDL-C, SBP, glucose and insulin. Non-significant increase was noted in DBP, TC and TG. |
| **Swift** (2018)  USA  RCT | **Mean age:** 52.4 ± 6.4  **Baseline mean BMI:** 29.7 ± 2.8  **Sample size:** 163  **Inclusion criteria:**  **BMI**: Overweight (BMI 25-29.9 kg/m^2^) and higher  **Co-morbidities:** Metabolic syndrome | **Lifestyle interventions:**   - Group 1: Low amount, moderate intensity exercise. - Group 2: Low amount, high intensity exercise. - Group 3: High amount, high intensity exercise. - Group 4: Non-exercise control group | 8 months | Intervention end | **Anthropometric**  WC  **Cardiovascular**  LDL-C, HDL-C, TG, TC, non-HDL-C, small LDL-C, large HDL-C, and LDL particles  **Metabolic**  Glucose, insulin, and HOMA-IR | Results were stratified by at least modest weight loss ≥3%, and no modest weight loss <3%. Examining all of the exercise groups together, 126 participants loss <3%. There was non-significant decrease in WC, insulin, HOMA-IR, TG, non-HDL-C, small LDL-C, and LDL particles. There was non-significant increase in glucose, LDL-C, HDL-C, TC, and large HDL-C. |
| **Thibault** (2015)  Canada  Secondary analysis of two prospective studies | **Mean age:** 57  **Baseline mean BMI (<5% weight loss group):** 34.4 ± 5.1  **Sample size:** 84  **Inclusion criteria:**  **BMI**: Overweight (BMI 25-29.9 kg/m^2^) and higher  **Co-morbidities:** Metabolic syndrome | **Lifestyle intervention:**   - Individual meetings with a nurse, a dietitian, and an endocrinologist every 6 weeks. - Seminars covering topics such as obesity, diet, exercise and behavior modification. | 12 months | Intervention end | **Anthropometric**  WC  **Metabolic**  Fasting glucose,  fasting insulin,  2h glucose post-OGTT, HbA1C | Results were stratified by weight loss ≥ 5%, and weight loss < 5%. Within the < 5% weight loss group, there was a significant decrease in WC, fasting glucose, 2h glucose post-OGTT and significant increase in fasting insulin and HbA1C. |
| **Tseng** (2002)  Taiwan  Intervention | **Mean age (SD):** 40.5 (12.3)  **Baseline mean BMI (SD):** 31.1 (3.8)  **Sample size:** 189  **Inclusion criteria:**  **BMI**: NA  **Co-morbidities:** NA | **Lifestyle interventions:**   - Behavioral weight loss program – nutrition lecture, group exercise sessions, meal preparation sessions and dietitian consult | 12 weeks | Intervention end | **Psychosocial and behavioral**  Brief Symptom Rating Scale, Bulimic Investigatory Test, Edinburgh | Results were stratified into weight loss <5%, ≥ 5%, and ≥ 10. For the participants who lost <5%, there was a significant improvement for the bulimic score, and a non-significant improvement for the general psychological score. |
| **Vasiljevic** (2012)  Serbia  Intervention | **Mean age (SD):** 41.8 (12.9)  **Baseline mean BMI (SD):** 36.2 (5.3)  **Sample size:** 135  **Inclusion criteria:**  **BMI**: Obesity class 1 (BMI 30-34.9 kg/m^2^) and higher  **Co-morbidities:** NA | **Lifestyle intervention**   - Dietitian led plan with check-ups every 3-4 weeks, hypocaloric diet, exercise instructions | 12 months | Intervention end | **Quality of life**  Impact of Weight on QOL-Lite | Results were stratified into ≥0% gain, 0.1–4.9% loss, 5.0–9.9% loss, ≥10.0% loss. For the 0.1-4.9% loss group, there was an improvement in the overall score (significance not tested). |
| **Vetter** (2013)  USA    RCT | **Mean age:** 51.5 ± 11.5  **Baseline mean BMI:** 38.5 ± 4.7  **Sample size:** 390  **Inclusion criteria:**  **BMI**: Obesity class 1 (BMI 30-34.9 kg/m^2^) and higher  **Co-morbidities:** Metabolic syndrome | **Lifestyle and pharmacological interventions:**   - Group 1: Usual Care, in which participants met quarterly with their PCP and received approximately 5–7 minutes of education about weight management. - Group 2: Brief Lifestyle Counseling (Brief LC), which included the quarterly PCP visit and 10–15 minutes of monthly behavioral counseling delivered by an auxiliary health care provider (typically a medical assistant) who was trained as a lifestyle coach. - Group 3: Enhanced Brief Lifestyle Counseling (Enhanced Brief LC), in which pharmacologic therapy (sibutramine or orlistat) or meal replacements were added to the same quarterly PCP visit and behavioral intervention to further enhance weight loss. | 24 months | 12 months, & intervention end | **Cardiovascular**  SBP, DBP, TG, TC, LDL-C, HDL-C, Mean non-HDL-C and TC/HDL-C ratio  **Metabolic**  Glucose, insulin, HOMA-IR  **Inflammatory biomarkers**  hs-CRP | Results were stratified by baseline weight or above, weight loss < 5%, weight loss ≥ 5 to < 10%, and weight loss ≥ 10%. Within the <5% weight loss group, SBP and DBP significantly increased at 12 and 24 months. Similarly, TC, TG, LDL-C, and hs-  significantly decreased. Glucose, HDL-C, and HOMA-IR significantly decreased at 12 months, however significantly increased at 24 months. |
| **Wing** (2010)  USA  RCT | **Mean age (SD):** 53 (11)  **Baseline mean BMI (SD):** 36 (6)  **Sample size:** 338  **Inclusion criteria:**  **BMI**: Overweight (BMI 25-29.9 kg/m^2^) and higher  **Co-morbidities:** Urinary incontinence | **Lifestyle interventions**   - Group 1: Behavioural weight loss program – weekly meetings months 1-6, fortnightly months 7-18, hypocaloric diet, physical activity - Group 2 (control): Structured education program – 7x 1 hour group education sessions (weight loss, physical activity, healthy eating) | 18 months | 6, 12, 18 months | **Quality of life**  Urinary incontinence per week (total, stress and urge);  24-hour involuntary urine loss;  Satisfaction with changes with incontinence | Results were stratified into gained weight, 0–<5% loss, 5.0–<10% loss, ≥10.0% loss. For the participants who lost 0-<5%, there was a decrease in the number of total incontinence episodes (including both stress and urge) and the amount of urine lost over 24 hours at all timepoints, compared to baseline. Across the three time points 62-64% of participants were satisfied with changes incontinence |
| **Wing** (2011)  USA  RCT | **Mean age:** 58.7 ± 6.8  **Baseline mean BMI:** 36.0 ± 5.9  **Sample size:** 5,145  **Inclusion criteria:**  **BMI**: Overweight (BMI 25-29.9 kg/m^2^) and higher  **Co-morbidities:** NA | **Lifestyle interventions:**   - Group 1: Decrease caloric intake and increase physical activity. - Group 2: Attended three meetings over the year and focused on diet, activity, and social support. | 12 months | Intervention end | **Cardiovascular**  SBP, DBP, TG, HDL-C, LDL-C  **Metabolic**  HbA1C, glucose | Results were stratified by gained >2%, stable gained ≤2 or lost <2, lost ≥2 to <5%, lost ≥5 to <10%, lost ≥10 to <15% and lost ≥15%. Those who lost 2–5% of their body weight had increased odds of having significant improvements in SBP, glucose, HbA1c and TG. Whereas DBP, HDL-C and LDL-C had increased odds of having improvements yet not significant. |
| **Wu** (2009)  Taiwan  Intervention | **Mean age:** 35.2 ± 1.0 years  **Baseline mean BMI:** 33.5 ± 0.4  **Sample size:** 119  **Inclusion criteria:**  **BMI**: NA  **Co-morbidities:** NA | **Lifestyle and pharmacological interventions:**   - Group 1: Low calorie diet suggestions (500 Kcal deficits per day) - Group 2: Low calorie diet suggestions + sibutramine (10 mg or 15 mg daily) - Group 3: Low calorie diet suggestions + orlistat (120 mg three times daily) - Group 4: Very low calorie diet regimens   All participants were encouraged to increase physical activity and frequency of exercise under the standard exercise instruction | 6 months | Intervention end | **Psychosocial and behavioral**  Physical functioning, role-physical, bodily pain, general health, vitality, social functioning, role-emotional, mental health, PCS scores, and MCS scores | Results were stratified by loss ≥ 15%, 10% ≤ weight loss < 15%, 5% ≤ weight loss < 10%, and weight loss < 5%. None of the outcomes presented a significant difference. However, a significant decrease in physical functioning was noted when adjusted for age and sex, compared the mean changes among groups with differing ranges of weight reduction. |

Adiponectin – APN, Area under the curve – AUC, Asthma health-related quality of life – AQLQ, Estimated glomerular filtration rate – eGFR, High-density lipoprotein cholesterol – HDL-C, C-Reactive Protein – CRP, High sensitivity C-Reactive Protein – hs-CRP, homeostasis model assessment of insulin resistance – HOMA-IR, low-density lipoprotein cholesterol – LDL-C, Fasting plasma glucose – FPG, Total cholesterol – TC, Triglycerides – TG, Tumor necrosis factor-alpha – TNF-α, Randomized controlled trial – RCT, United States of America – USA, Monocyte chemoattractant protein-1 – MCP-1, Waist circumference – WC, Systolic blood pressure – SBP, Diastolic blood pressure – DBP, Left-ventricle ejection fraction – LVEF, New York Heart Association – NHYA, Brain natriuretic peptide – BNP, Oral glucose tolerance test – OGTT, Physical Component Summary – PCS, Mental component summary – MCS, Aspartate aminotransferase – AST, Alanine aminotransferase – ALT, Gamma-glutamyl transpeptidase – γGTP, gamma-glutamyl transferase – GGT, Forns fibrosis index – FI, AST to platelet ratio index – APRI, Forced expiratory volume in one-second – FEV1, Forced vital capacity – FVC, Total lung capacity – TLC, Inspiratory capacity – IC, Functional residual capacity – FRC, Expiratory reserve volume – ERV, Residual volume – RV, Asthma control questionnaire – ACQ, Asthma-related quality of life questionnaire – AQLQ, Apnea-hypopnea index – AHI, Apnea index – AI, Nonrapid eye movement sleep – NREM-AHI Rapid eye movement sleep – REM-AHI, Hypopnea index – HI, Oxygen desaturation index – ODI, Oxygen saturation – SaO2, Non-alcoholic fatty liver disease – NAFLD, fatty liver index – FLI, New York Heart Association scale – NYHA, Serum amyloid A – SAA, Angiotensinogen – AGT, Malondialdehyde – MDA
